# Supplementary figures and images for: An elasticity-curvature illusion decouples cutaneous and proprioceptive cues in active exploration of soft objects
Source: PLoS Comput Biol. 2021 Mar 22;17(3):e1008848. doi: 10.1371/journal.pcbi.1008848 (PMC8016306; doi:10.1371/journal.pcbi.1008848)

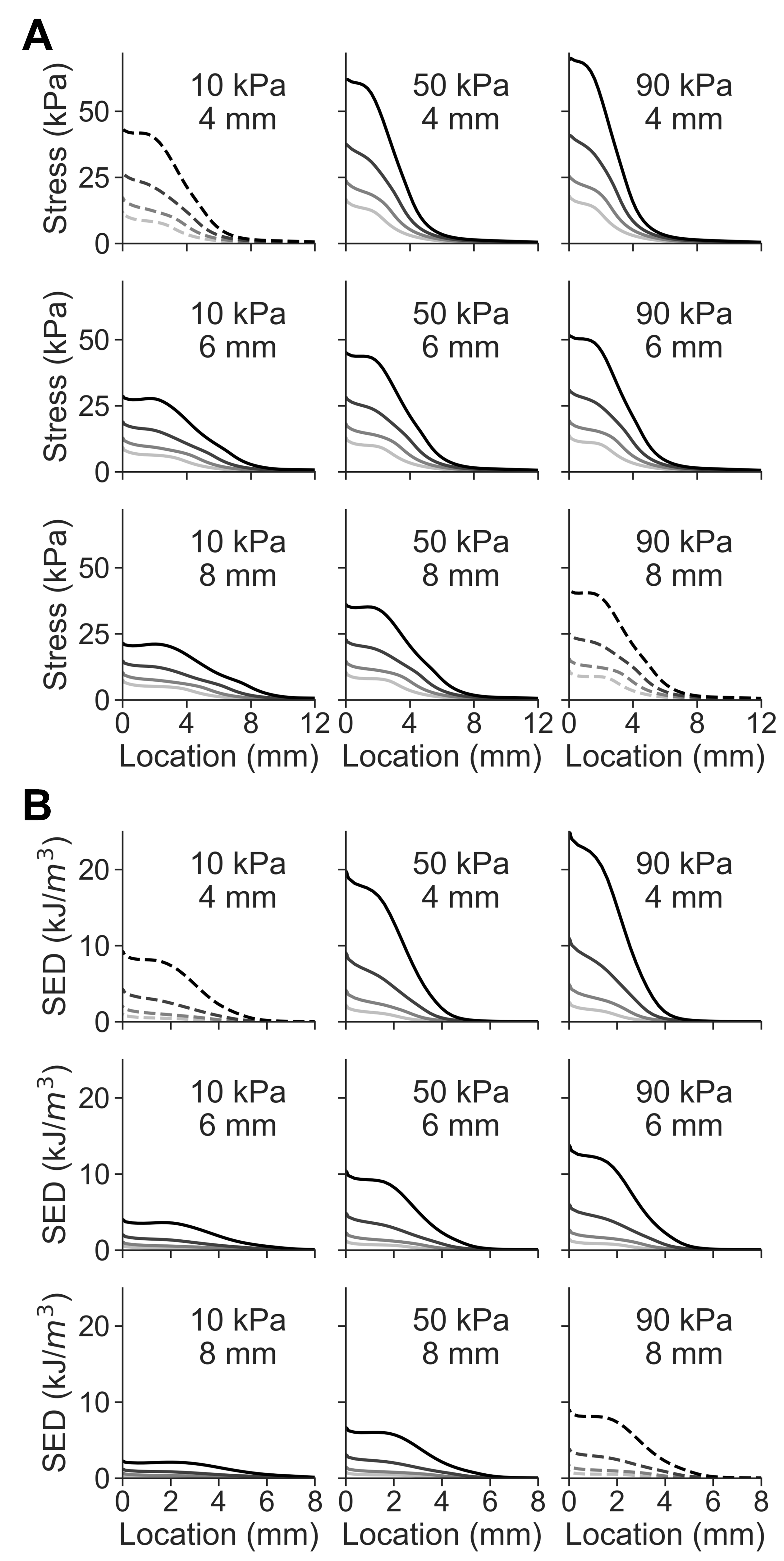

Supplement: S1 Fig — (A) Spatial distributions of stress at contact locations for all nine spherical stimuli. (B) Spatial distributions of SED at the same contact locations for all spheres varying in radii and elasticity. (TIF) [file pcbi.1008848.s001.tif]

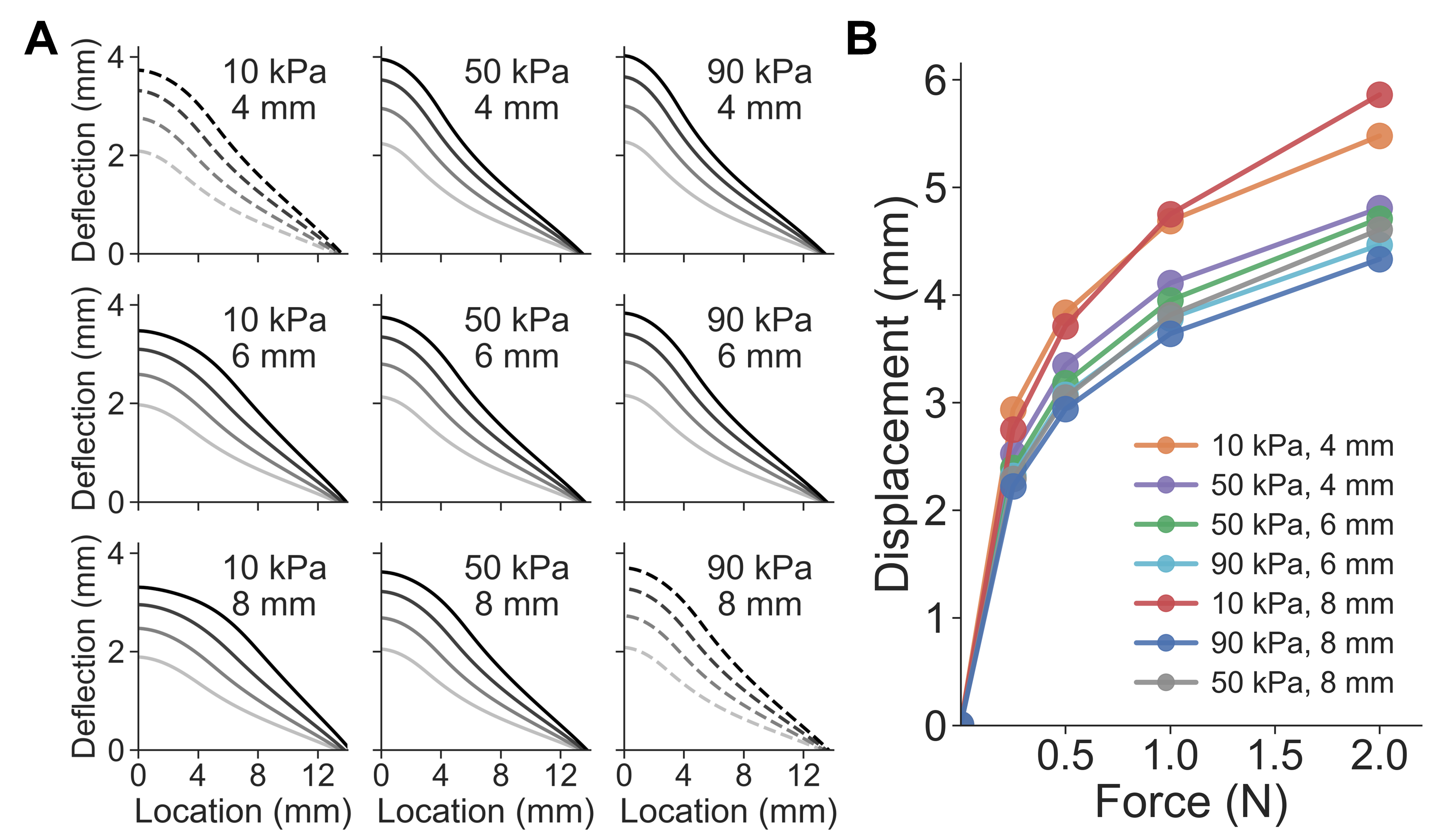

Supplement: S2 Fig — (A) Simulated surface deflection of nodes at the surface of the finger pad model for all the nine spheres. (B) Force-displacement relationships of the fingertip simulated for elasticity-radius combinations. (TIF) [file pcbi.1008848.s002.tif]

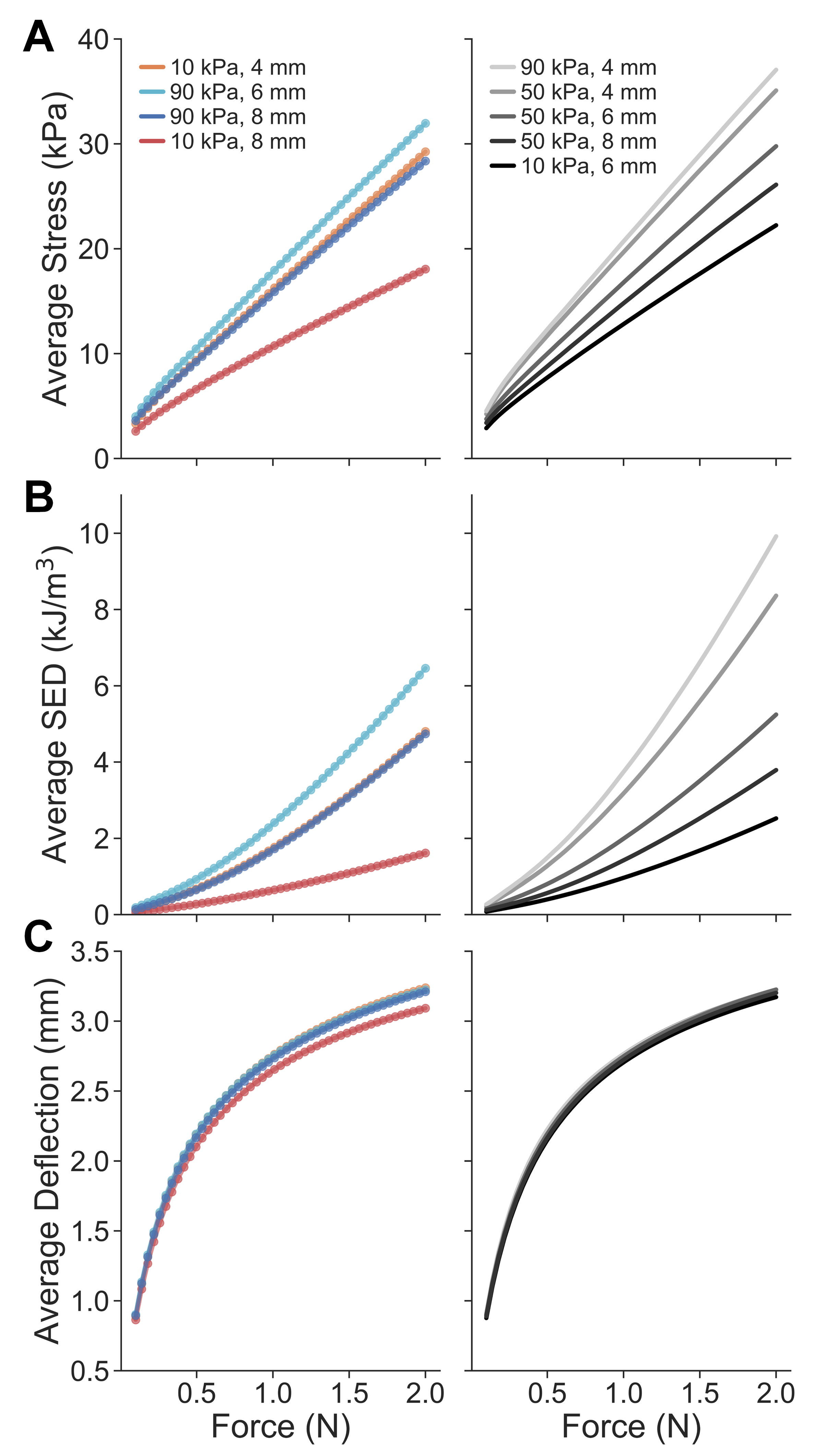

Supplement: S3 Fig — For the intermediate force loads, average responses were quantified over the same contact region for tactile cues of (A) stress, (B) SED, and (C) surface deflection. The average stress/strain distributions overlap for the illusion case spheres, while similar average deflection cues were derived from all nine stimuli. (TIF) [file pcbi.1008848.s003.tif]

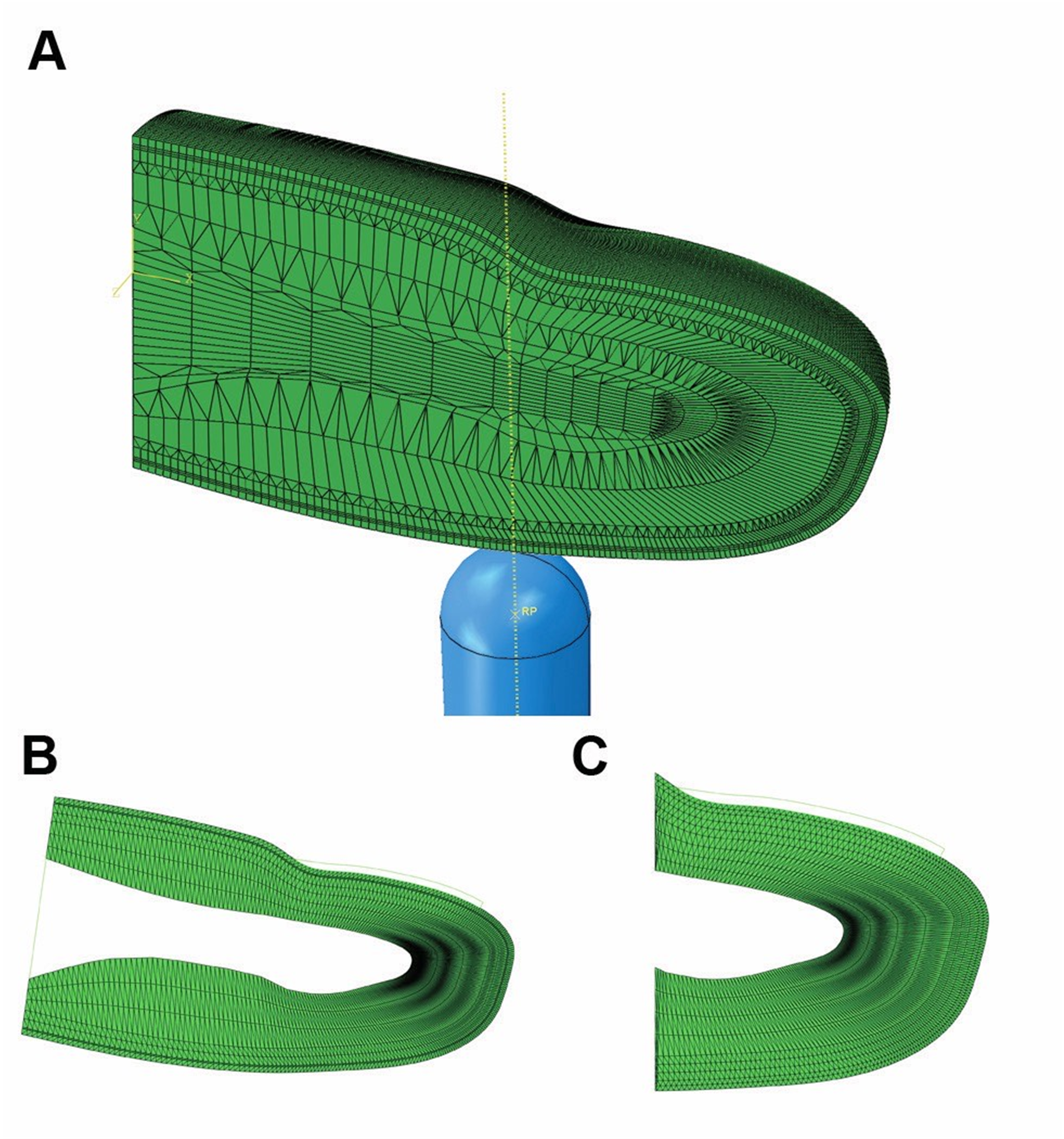

Supplement: S4 Fig — (A) The compliant stimulus is implemented as hemispheres contacting the skin surface of the finger pad. (B) Plane-strain model to fit the surface deflection. (C) Axisymmetric model to fit force-displacement relation and perform simulations. Adapted from [35] with permission. (TIF) [file pcbi.1008848.s004.tif]

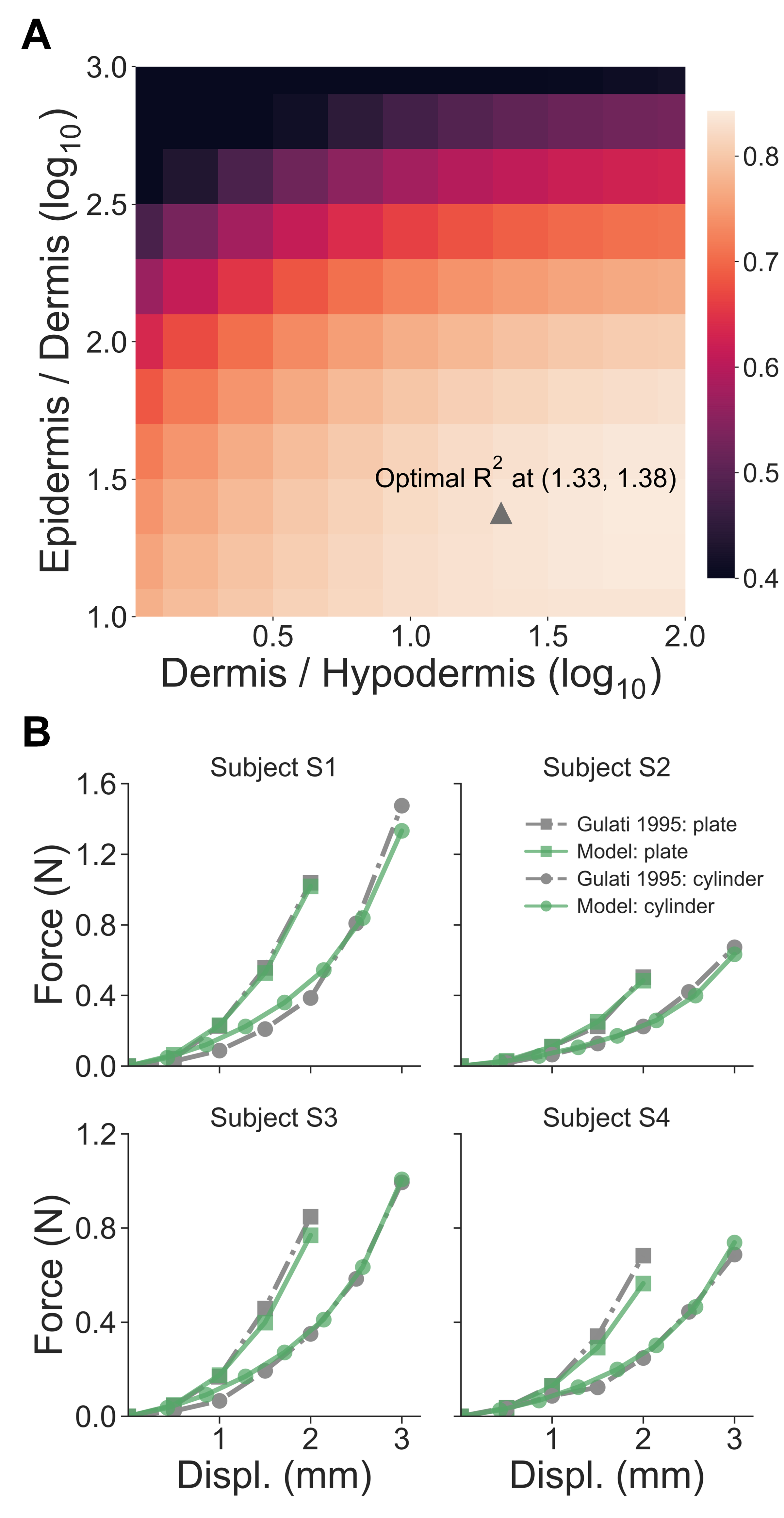

Supplement: S5 Fig — (A) Relative ratios between skin layers are optimized to fit the surface deflection simulated by the model. The optimal point is selected by averaging all points with a R2 ≥ 0.8. (B) Force-displacement fits between model simulations and experimental measurements. Adapted from [35] with permission. (TIF) [file pcbi.1008848.s005.tif]

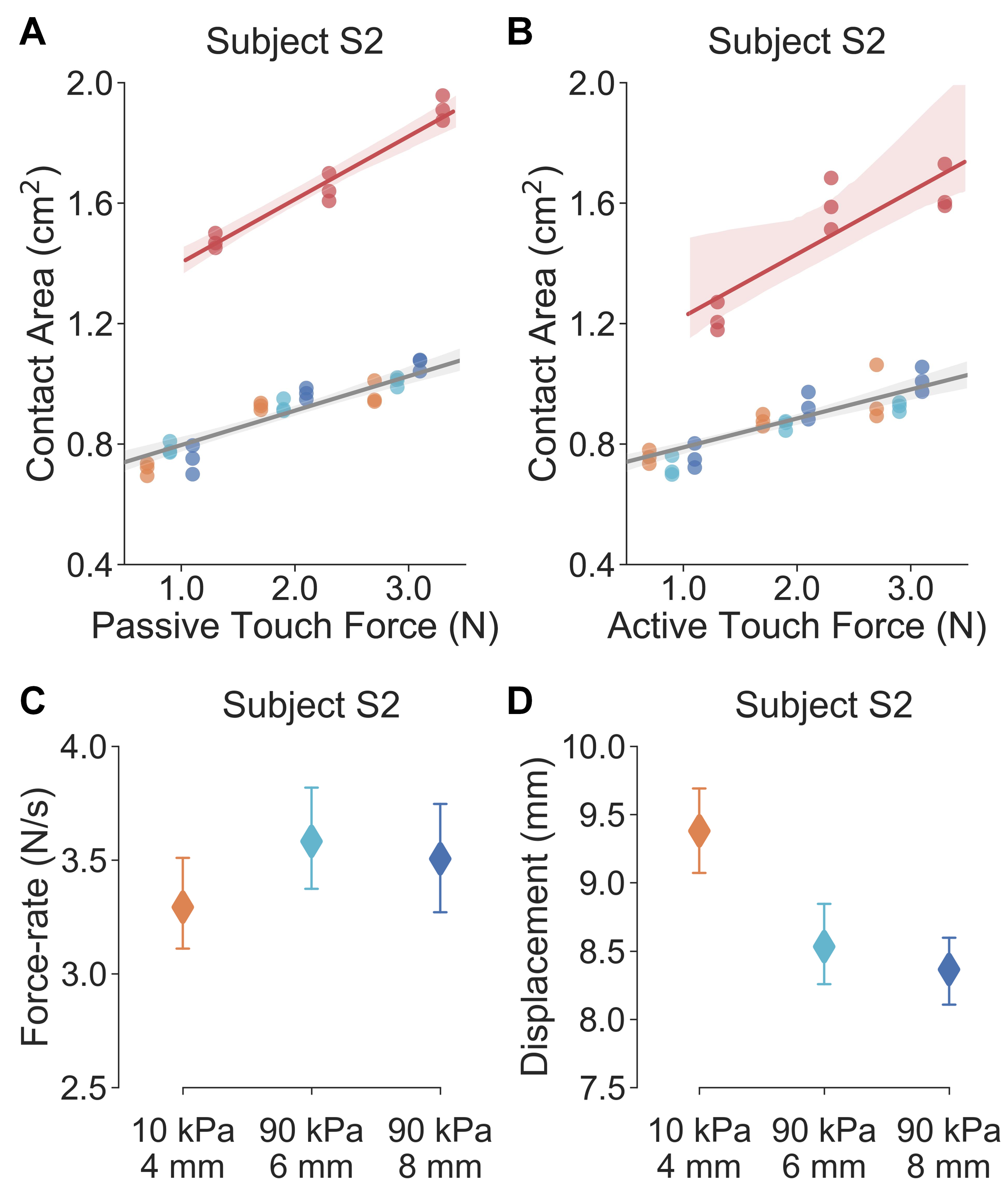

Supplement: S6 Fig — Gross contact areas measured in (A) passive and (B) active touch from one representative participant. Linear regression procedures are applied to visualize the correlation between touch force and contact area. Translucent bands denote 95% confidence intervals for regression estimations. (C) Similar force-rates are volitionally controlled and applied in active exploration of illusion case spheres. (D) Distinct fingertip displacements are applied in discriminating the illusion case spheres. (TIF) [file pcbi.1008848.s006.tif]

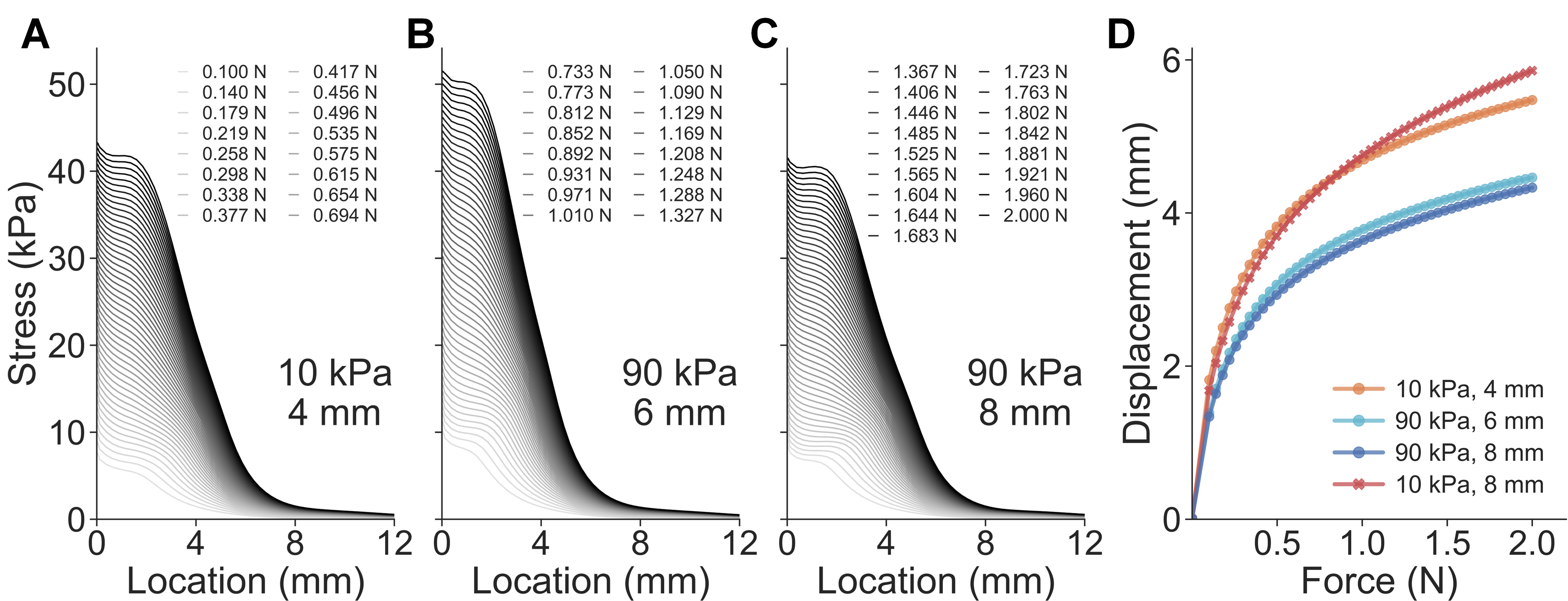

Supplement: S7 Fig — Stress distributions at contact locations for the three illusion case spheres: (A) 10 kPa-4 mm, (B) 90 kPa-6 mm, and (C) 90 kPa-8 mm. (D) Proprioceptive cues of finger displacement are simulated for all discretized force load during the ramp phase. (TIF) [file pcbi.1008848.s007.tif]

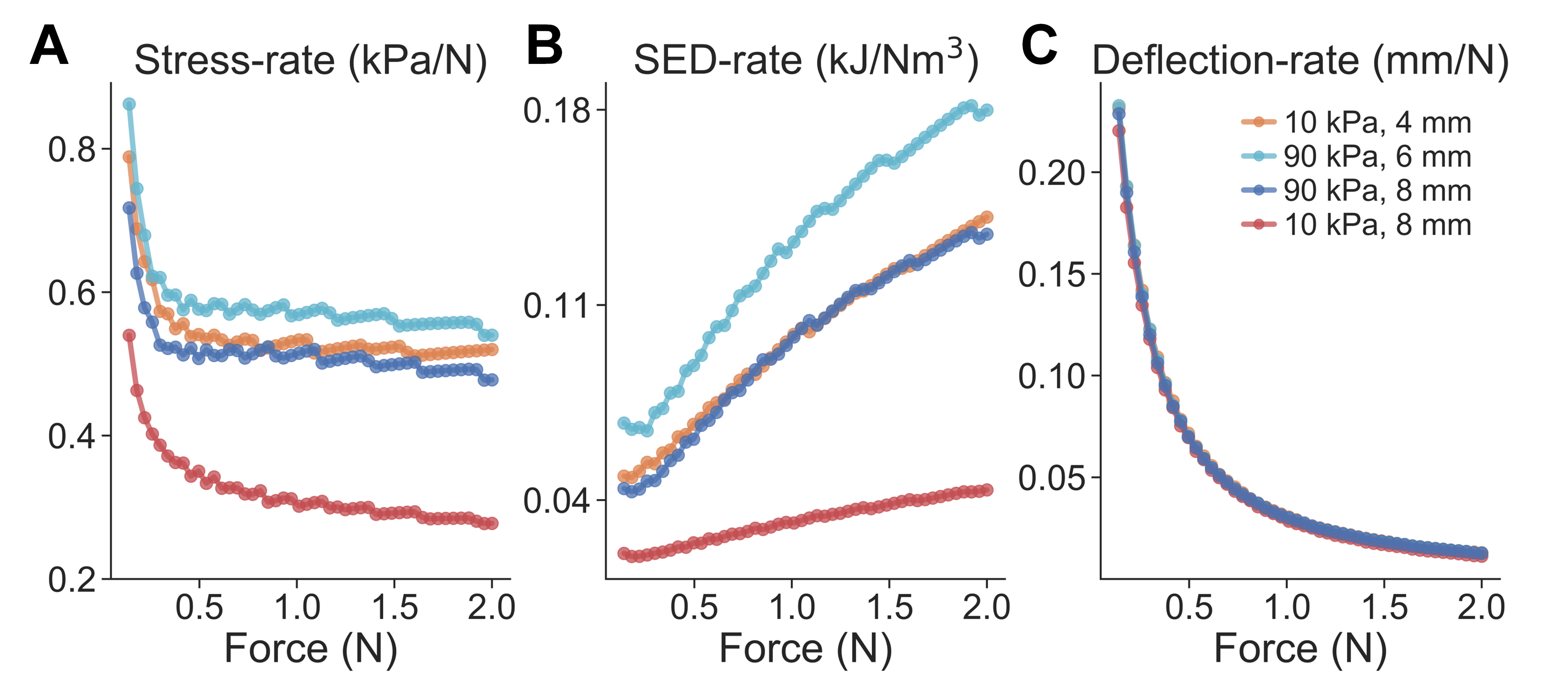

Supplement: S8 Fig — Derived from S3 Fig, the rate of change of averaged (A) stress, (B) SED, and (C) surface deflection are calculated for the contact ramp phase. Note that within the simulation procedure, time points are linearly coupled with force loads, i.e., 0.5 N is applied at 0.25 sec and 1.5 N is applied at 0.75 sec, etc. (TIF) [file pcbi.1008848.s008.tif]
