# Supplementary material for: An elasticity-curvature illusion decouples cutaneous and proprioceptive cues in active exploration of soft objects
Source: PLoS Comput Biol. 2021 Mar 22;17(3):e1008848. doi: 10.1371/journal.pcbi.1008848 (PMC8016306; doi:10.1371/journal.pcbi.1008848)
Supplement: S1 Text — The text includes five sections: perceptual cues predicted in the computational modeling, geometry of the fingertip model, fitting hyperelastic material properties, perceptual cues measured from one representative participant, and perceptual cues predicted during the dynamic contact. (DOCX) [file pcbi.1008848.s013.docx]

**Supporting information for**

An elasticity-curvature illusion decouples cutaneous and proprioceptive cues in active exploration of soft objects

Short title: A tactile illusion for softness perception

Chang Xu^1^, Yuxiang Wang^1^, Gregory J. Gerling^1^*

^1^School of Engineering and Applied Science, University of Virginia, Charlottesville, Virginia, United States of America.

* [gg7h@virginia.edu](mailto:gg7h@virginia.edu)

**Perceptual cues predicted in the computational modeling**

For the cutaneous-only cues, besides spatial distributions of stress, other response variables were also derived from the finger-stimulus contact mechanics. Strain energy density (SED) at the epidermal-dermal interface where Merkel cell end-organs of slowly adapting type I (SAI) afferents reside were estimated by averaging neighboring elements at each interface node. As illustrated in S1 Fig, similar distributions were obtained for the cue of stress/strain across all spheres. Either an increase of the elasticity or a decrease of the sphere radius will increase the concentration of stress/strain at contact locations, with the lowest for the 10 kPa-8 mm sphere and the highest for the 90 kPa-4 mm sphere.

Furthermore, the variations in radius counteracted the changes in elasticity, resulting in nearly identical stress/strain distributions at the same contact locations. As illustrated in Figs 3 and S1, compared with the distinct combination of 10 kPa-4/8 mm, overlapping curves were obtained for small-compliant (10 kPa-4 mm) and large-stiff spheres (90 kPa-8 mm). In addition, curves for the 10 kPa-4 mm and 90 kPa-6 mm spheres were fairly similar. These results were consistent with spatial distributions of stress shown in Figs 2 and S1.

Besides the stress/strain cue at the locations of mechanoreceptive end organs, deflection of the skin surface is often considered as a cutaneous cue informing the change of contact area [1,2]. Specifically, deflection of the skin’s surface is the contour of a deflection profile in the contact plane, readily obtainable by visual observation through sophisticated cameras [3,4]. Therefore, displacements at the node of the epidermis surface were calculated as the skin deflection cue. Similar to results of stress/strain distributions, overlapping curves were obtained from the same stimuli pair (i.e., 10 kPa-4 mm and 90 kPa-8 mm, Fig 3C). Additionally, as in Fig 3D, deflection curves for the 10 kPa-4 mm and 90 kPa-6 mm spheres were inseparable, which were predicted to generate nearly identical contact area cues. Overall, as illustrated in S2A Fig, an increase in the elasticity or a decrease in the spherical radius will increase the magnitude of the surface deflection. Consistent trends were obtained for stress and SED distributions (S1 Fig).

For the proprioceptive cue simulated for active touch, fingertip displacement was derived from the translational movement of the fingertip bone in the normal direction. In general, as illustrated in S2B Fig, an increase in the radius or the elasticity will contribute to a decrease in the fingertip displacement given the same loading force.

Based on the analysis of stress/strain distributions and surface deflection, average responses of these contact cues were calculated to further quantify the similarity/difference among the illusion case spheres (10 kPa-4 mm, 90 kPa-6 mm, and 90 kPa-8 mm), the distinct sphere (10 kPa-8 mm), and all the others. In particular, for all element nodes within 6 mm at the contact interface, i.e., the location range is [0, 6] mm, the mean response of each contact cue was calculated at each force load (S3 Fig).

Compared with the other six spheres, we found that the illusion case spheres generate overlapped stress/strain responses. However, similar surface deflection results were observed upon contact for all nine stimuli. Specifically, compared with the distinct sphere (10 kPa-8 mm), overlapping stress/strain-force curves were obtained for the three illusion case spheres (S3 Fig). The average stress distributed over the contact region was 17.15 ± 7.70 kPa (mean ± SD) for three illusion case spheres across all force loads, as compared to the distinct sphere of 10.88 ± 4.48 kPa. Likewise, the average SED was 2.31 ± 1.63 kJ/m^3^ for the illusion case spheres across all force loads, as compared to the distinct sphere of 0.74 ± 0.47 kJ/m^3^. Last, the average surface deflection across force loads was 2.59 ± 0.59 mm for the illusion case spheres, as compared to the distinct sphere of 2.49 ± 0.57 mm.

**Geometry of the fingertip model**

As illustrated in S4A Fig, the fingertip-stimulus contact and movement were simulated with finite element models of the human distal phalange and compliant spheres. Two 2D models were built to characterize the geometry and material of the fingertip [5]. As shown in S4B Fig, the plane-strain model was created from a cross-sectional slice from proximal first digit to distal tip, to account for stimuli contacting across the width of the finger. The axisymmetric model was built to analyze the stimuli contact mechanics normal to the contact surface (S4C Fig).

The finger bones and nails were modeled as rigid bodies as they were much stiffer than soft tissues. Three layers of soft tissues were included as deformable bodies, wrapping around the finger bone, namely epidermis, dermis, and hypodermis. There were no relative displacements among layer interfaces. The fingernail was modeled as 13 mm in length and 0.46 mm in thickness.

Near the surface of the finest dimension, the mesh was built with 0.25 mm wide elements. Gradually, larger sizes were used closer to the finger bone. Triangular meshes were used both in the plane-strain and axisymmetric models.

**Fitting hyperelastic material properties**

In general, the plane strain model (S4B Fig) was first utilized to fit material properties of the surface deflection, and the axisymmetric model (S4C Fig) was then used to fit force-displacement relations and further perform simulations with compliant stimuli.

In the first step, material properties of the innermost layer (i.e., hypodermis or subcutaneous tissue) were predefined, and only the initial shear modulus of the epidermis and dermis were adjusted to calibrate the relative ratios between the elasticity of each layer. Since surface deflection is independent of the absolute material properties and is only controlled by relative ratios between layers, deflection data from prior in vivo experiments were used to fit all the ratios in our model.

Specifically, the initial shear modulus of the hypodermis layer was set to be 1 kPa, as similar to Wu *et al*. [6]. The reasonable search range for dermal elasticity was set as 1 to 100 times of the hypodermis, and for epidermal elasticity was 10 to 1000 times of the dermis. An exhaustive fitting procedure was applied with a step size of 0.2 on a common-log scale within this search range (S5A Fig). Two rigid body indenters were used herein, both rigid cylindrical indenters with diameter of 3.17 and 9.52 mm, and both employing the plane strain model. Surface deflection was first simulated at six displacements (0.5, 1.0, 1.6, 2.5, 3.0, and 3.5 mm) with candidate ratios and then compared with the in vivo experimental data from Dandekar [7]. The average of the coordinates of all points with a *R^2^* ≥ 0.8 were then calculated as the optimal ratios (S5A Fig). The final modulus ratio between the epidermal, dermal, and hypodermal layers was 510.63: 21.37: 1.00.

In the second step, based on fitted ratios and surface deflections, the elastic moduli of the materials were scaled to fit the displacement-force data measured for four subjects in prior experiments [8]. Two rigid indenters were used to simulate the displacement-force responses in the axisymmetric model: a cylinder of 6.35 mm diameter, and a flat plate. By optimizing the average *R^2^* for each indenter per each subject using the L-BFGS-B algorithm, the optimal scaling for each material layer was determined. As illustrated in S5B Fig and detailed in S1 Table, the final *R^2^* was 0.99 and final shear moduli were 1.21 MPa for epidermis, 50.67 kPa for dermis and 2.37 kPa for hypodermis [5]. This result is comparable to prior data by Wu *et al*. [6].

**Perceptual cues measured from one representative participant**

In the biomechanical measurement experiments, gross contact areas were measured using the ink-based procedure in both passive and active touch. As illustrated in S6A-B Fig, in both interaction conditions, the illusion case spheres (i.e., 10 kPa-4 mm, 90 kPa-6 mm, and 90 kPa-8 mm) indeed generate similar contact areas, as opposed to the distinct stimulus 10 kPa-8 mm, which generated significantly higher contact areas.

Furthermore, a strong linear correlation was verified between touch force and contact area. In passive touch (S6A Fig), the Spearman’s rank coefficient yielded linear correlations of 0.90 (*p* = 2.43*e*^-10^) and 0.95 (*p* = 9.58*e*^-5^) for the illusion and distinct spheres respectively. In active touch (S6B Fig), the Spearman’s rank coefficient yielded linear correlations of 0.89 (*p* = 4.67*e*^-10^) and 0.84 (*p* = 0.004) for the illusion and distinct spheres respectively.

This indicates that within the designated force range, the change of contact area, elicited by the touch interaction, was proportional to the touch force. Since the force-rate was linearly controlled in passive touch, the change in contact area could be directly quantified by the force-rate cue within current measurement limitations. This results also explained that the force-rate cue induced in passive touch indeed elicited the change of contact area, which exhibited a strong linear correlation and is consistent with prior work [9,10].

**Perceptual cues predicted during the dynamic contact**

To further investigate how contact with the illusion case spheres might impact afferent responses during the dynamic contact phase, especially for rapidly adapting mechanoreceptors, we simulated the ramp phase by interpolating loads and derived response variables with higher precision. In particular, we evenly inserted 48 more time points in the simulation procedure and thus, interpolated the force load of 0 to 2 N with total 50 discretized intermediate loads to fully simulate dynamic contact. These discretized loads are shown in S7 Fig as legends. Note that the first load (0 N) is omitted.

As illustrated in S7A-C Fig, across all intermediate force loads, the illusion case spheres (i.e., 10 kPa-4 mm, 90 kPa-6 mm, and 90 kPa-8 mm) indeed generated similar stress distributions at contact locations. Specifically, stress curves of the 10 kPa-4 mm and 90 kPa-8 mm nearly overlapped. Based on these model predictions, these results indicate that, during the dynamic contact phase, i.e., early ramp stage, afferent responses that might be elicited given a stress input would be expected to be nearly consistent among the illusion case spheres.

To quantify the similarity of cutaneous cues from illusion case spheres, averaged responses (stress/SED/surface deflection) over a contact region were calculated (S3 Fig). Compared with the other six spheres, we found that illusion case spheres generate overlapped stress/strain/deflection responses over the whole contact time-course. Furthermore, the rate of change of these cutaneous cues consistently overlap (S8 Fig). These indicate that upon dynamic contact the illusion case spheres might be expected to elicit similar afferent responses from rapidly adapting mechanoreceptors. Therefore, it is likely that compliances of the illusion case spheres are consistently indiscriminable during the stimulus ramp.

**References**

1. Moscatelli A, Bianchi M, Serio A, Terekhov A, Hayward V, Ernst MO, et al. The change in fingertip contact area as a novel proprioceptive cue. Curr Biol. 2016;26: 1159–1163. doi:10.1016/j.cub.2016.02.052

2. Xu C, Wang Y, Hauser SC, Gerling GJ. In the tactile discrimination of compliance, perceptual cues in addition to contact area are required. Proc Hum Factors Ergon Soc Annu Meet. 2018;62: 1535–1539. doi:10.1177/1541931218621347

3. Dzidek B, Bochereau S, Johnson SA, Hayward V, Adams MJ. Why pens have rubbery grips. Proc Natl Acad Sci U S A. 2017;114: 10864–10869. doi:10.1073/pnas.1706233114

4. Nam S, Vardar Y, Gueorguiev D, Kuchenbecker KJ. Physical variables underlying tactile stickiness during fingerpad detachment. Front Neurosci. 2020;14: 235. doi:10.3389/fnins.2020.00235

5. Wang Y, Gerling GJ. Computational modeling reinforces that proprioceptive cues may augment compliance discrimination when elasticity is decoupled from radius of curvature. In: Auvray M, Duriez C, editors. Haptics: Neuroscience, Devices, Modeling, and Applications. Berlin, Heidelberg: Springer Berlin Heidelberg; 2014. pp. 360–368.

6. Wu JZ, Dong RG, Rakheja S, Schopper AW, Smutz WP. A structural fingertip model for simulating of the biomechanics of tactile sensation. Med Eng Phys. 2004;26: 165–175. doi:10.1016/j.medengphy.2003.09.004

7. Dandekar K. Role of mechanics in tactile sensing of shape. Massachusetts Institute of Technology. 1995.

8. Gulati RJ, Srinivasan MA, others. Human fingerpad under indentation I: static and dynamic force response. ASME-Publications-Bed. 1995;29: 261.

9. Xu C, He H, Hauser SC, Gerling GJ. Tactile exploration strategies with natural compliant objects elicit virtual stiffness cues. IEEE Trans Haptics. 2020;13: 4–10. doi:10.1109/TOH.2019.2959767

10. Hauser SC, Gerling GJ. Force-rate cues reduce object deformation necessary to discriminate compliances harder than the skin. IEEE Trans Haptics. 2018;11: 232–240. doi:10.1109/TOH.2017.2715845
