# Supplementary material for: An elasticity-curvature illusion decouples cutaneous and proprioceptive cues in active exploration of soft objects
Source: PLoS Comput Biol. 2021 Mar 22;17(3):e1008848. doi: 10.1371/journal.pcbi.1008848 (PMC8016306; doi:10.1371/journal.pcbi.1008848)
Supplement: Attachment — Submitted filename: ResponseToReviewers_PCOMPBIOL-D-20-01703-Responses.pdf [file pcbi.1008848.s014.pdf]

Reviewer #1 Comments

General comments:

The authors describe a set of experiments in which they have fabricated a set of stimuli whose elasticity and curvature vary so as to explore the contact mechanics between the objects and the finger and the perceived softness of the objects. Computational models of the contact mechanics between the fingertip and spheres of varying radii and elasticity were initially developed to capture the stress distributions and finger surface deflection during contact. Based on these models, particular stimuli were selected for the biomechanical and psychophysical experiments. The results are of interest in that they demonstrate the critical role of proprioceptive cues in perceiving the compliance of manually explored objects and of the rate of change in contact area when objects are sensed under passive touch conditions.

One major issue with the manuscript is how the models developed and experiments conducted are framed. The authors refer to their development of an illusion which is not an appropriate approach to understanding human perception. A perceptual illusion refers to a misperception or a discrepancy between a physical stimulus and its corresponding percept. It is not that an illusion is developed a priori, more that a set of stimuli are fabricated (by varying elasticity and curvature in this case) that have particular properties based on the FEMs of the fingerpad and object that could result in errors in perception. Once these "errors" have been demonstrated, that is that there is an illusion, the focus is on understanding the mechanisms that account for the perceptual errors. By analogy to the size-weight illusion that the authors refer to, the stimuli designed for experiments studying this illusion are not by definition illusory, volume and mass are covaried to create a stimulus set. Similarly, the term "illusory cutaneous contact" does not accurately depict the mechanics of finger contact which are not illusory.

The authors propose that because the spheres are indiscriminable when explored passively they create an illusory experience and that when explored actively the illusion disappears. The "illusion" is not a property of the spheres but of how they are explored. With active touch there is no illusion and so it does not seem to make sense to refer to these spheres as illusory. Similarly, with the passive direct-force rate presentation the spheres were now discriminable.

> We appreciate the reviewer's very well-articulated perspective. The reviewer delineates that the illusion represents a discrepancy between a physical stimulus and its corresponding percept, as noted elsewhere [1], and we have worked to reframe the manuscript's language accordingly. We have modified text describing the computational modeling and empirical experiments, particular stimuli, and mechanisms involved in integrating underlying sensorimotor signals.

1. Lederman SJ, Jones LA. Tactile and haptic illusions. IEEE Trans Haptics. 2011;4: 273–294. doi:10.1109/TOH.2011.2

> Furthermore, the reviewer notes the role of this illusion (and study) to "*demonstrate the critical role of proprioceptive cues in perceiving the compliance of manually explored objects and of the rate of change in contact area when objects are sensed under passive*

touch conditions." This statement also well articulates what we had intended to communicate, and we have revised the manuscript to improve its expression.

We have made revisions throughout the manuscript. Some of the revisions can also be found in responses to the reviewer's comment #5, 6, 7, 13, and 17.

Revisions in: Abstract

The old sentence: …we develop a tactile illusion to decipher the percept of softness, where small-compliant and large-stiff spheres are indiscriminable.
[p.2 line 17] The new sentence: …we investigate an illusion phenomenon in exploring softness; where small-compliant and large-stiff spheres are indiscriminable.

The old sentence: By modulating contact mechanics at the finger pad, we find this elasticity-curvature illusion is observable in passive touch, …
[p.2 line 18] The new sentence: By modulating contact interactions at the finger pad, we find this elasticity-curvature illusion is observable in passive touch, …

The old sentence: We subsequently exploit this illusion to dissociate relative contributions from cutaneous and proprioceptive signals…
[p.2 line 22] The new sentence: We subsequently exploit this phenomenon to dissociate relative contributions from cutaneous and proprioceptive signals…

The old sentence: …by controlling surface contact force to optimally elicit and integrate proprioceptive inputs amidst illusory cutaneous contact.
[p.2 line 24] The new sentence: …by controlling surface contact force to optimally elicit and integrate proprioceptive inputs amidst indiscriminable cutaneous contact.

The old sentence: …we find the illusory spheres can be rendered discriminable when…
[p.2 line 26] The new sentence: …we find those spheres are discriminable when…

Revisions in: Author summary

The old sentence: This study identifies an important natural illusion that deciphers our perception of softness.
[p.2 line 30] The new sentence: This study investigates an illusion phenomenon that occurs in discriminating material compliances.

The old sentence: We develop small-compliant and large-stiff spheres that are naturally indistinguishable when pressed into…
[p.2 line 31] The new sentence: We find that small-compliant and large-stiff spheres are naturally indistinguishable when pressed into…

The old sentence: This illusion illuminates an interplay within our somatosensory system, in particular, between cutaneous response from skin.…
[p.2 line 33] The new sentence: This phenomenon illuminates an interplay within our somatosensory system, in particular, between cutaneous responses from skin…

Revisions in: Results

[p.5 line 95] New sentences: …to develop elasticity-curvature combinations that afford non-differentiable cutaneous cues. Then, investigation of the mechanisms that underlie this potential illusory experience is done empirically with human-subjects via measurements of biomechanical interactions and evaluations of psychophysical responses. The results suggest…

Revisions in: Experiment 3: psychophysical evaluation of the elasticity-curvature illusion

The old sentence: To validate whether these compliant spheres might indeed render a perceptual illusion, we conducted…

[p.12 line 257] The new sentence: To evaluate whether there is a perceptual illusion in exploring these compliant spheres, we conducted…

Revisions in: Discussion

[p.14 line 319] New sentences: This study investigates an illusion phenomenon in exploring soft objects, specifically where small-compliant and large-stiff spheres are indiscriminable. These two physical attributes are common to everyday tasks; for example, in judging the ripeness of fruit. …we show that small-compliant and large-stiff spheres afford nearly identical cutaneous contact, and thus, are indiscriminable in passive touch where only cutaneous cues are available.

Detailed corrections:

1. p.3 line 36: cutaneous response – responses

Revisions in: Author summary

The old sentence: …between cutaneous response from skin receptors…

[p.2 line 34] The new sentence: …between cutaneous responses from skin receptors…

2. p.3 line 57: With it – what does "it" refer to?

We changed the text to clarify the confusion.

Revisions in: Introduction

[p.3 line 55] New sentences: Another intriguing illusion regards our perception of curvature where a physically flat surface is manually explored along a lateral direction. Depending on the relative inward/outward motions of the surface and the observer's finger, the flat surface could be perceived as being convex or concave curved [9,14].

3. p.4 line 61: in analogy – by analogy.

Revisions in: Introduction

The old sentence: A further illusion, in analogy with the Aubert-Fleischl…

[p.4 line 60] The new sentence: A further illusion, by analogy with the Aubert-Fleischl…

4. **p.4 lines 62-63: the tactile/proprioceptive illusion described here should have more detail so that the reader can understand the effect – the speed of a moving stimulus changes depending on whether it is perceived via tactile motion or with arm movement.**

   We made corresponding changes to better illustrate this illusion case.

Revisions in: Introduction

[p.4 line 60] New sentences: …indicates a possible misperception in the speed of a moving stimulus by touch [15]. Compared with tracking the stimulus with a guided hand movement (i.e., proprioception is available), observers can overestimate the stimulus speed with a stationary hand (i.e., tactile cues only).

5. **p. 4 line 77: In what way does the tactile illusion "underlie our perception of softness" which implies that the illusion is fundamental to perception?**

   We agree with the reviewer that this language is not precise. We have revised this section to better frame this point.

Revisions in: Introduction

[p.5 line 82] The new paragraph: Here, we investigate a tactile illusion associated with softness perception, specifically, in exploring spherical stimuli with covaried elasticity and curvature. These physical attributes are routinely encountered, such as in judging the ripeness of spherical fruit…

6. **p. 5 line 82: If the illusion is observed only under passive touch conditions how does it "naturally decouple cutaneous cues from proprioceptive movements" given the latter do not occur with passive touch?**

   To be more accurate, we find this illusory experience is only observed under passive touch. However, in active touch, those stimuli still afford similar (not significantly different) cutaneous cues (terminal contact area, stress/strain distributions, and surface deflection), but distinct proprioceptive cues (fingertip displacements). Therefore, these stimuli could dissociate relative contributions of cutaneous and proprioceptive signals in encoding softness perception. We have changed the text below to better clarify this.

Revisions in: Introduction

[p.5 line 85] New sentences: …when the finger is stationary and only non-distinct cutaneous cues are available for perception. The spheres, however, become readily discriminable when explored volitionally in active touch where finger proprioception is involved. The spheres therefore naturally dissociate relative contributions from cutaneous and proprioceptive cues in encoding softness, and shed light into…

7. **p. 5 lines 85-86: Is it really correct to state that "small-compliant and large-stiff spheres are perceived as identical?" The fact that participants cannot discriminate reliably between them does not mean they are perceived as identical. The results show that they cannot be discriminated.**

   Indeed, they're differentiable, not identical, and we have changed the language. The psychophysical results showed that these stimuli cannot be discriminated under the "passive same fore-rate" and "passive inverse force-rate" experimental conditions.

Revisions in: Results

The old sentence: …elasticity-curvature illusion where small-compliant and large-stiff spheres are perceived as identical.

[p.5 line 91] The new sentence: …elasticity-curvature illusion where small-compliant and large-stiff spheres are perceived as indiscriminable in passive touch.

The old sentence: …small-compliant (10 kPa–4 mm) and large-stiff (90 kPa–8 mm) spheres will generate nearly identical cutaneous contacts, …

[p.5 line 102] The new sentence: …small-compliant (10 kPa–4 mm) and large-stiff (90 kPa–8 mm) spheres will generate nearly identical cutaneous contact cues, …

8. **p. 5 line 86: "This is done for single, bare-finger touch" – What is done?**

   To explore the softness of these spheres, participants were instructed to use single, bare finger to touch the stimuli under both passive and active touch conditions. We changed the text to better clarify this point.

Revisions in: Results

The old sentence: This is done for single, bare finger touch.

[p.5 line 92] The new sentence: These spheres are explored using single, bare finger touch.

9. **p. 12 line 216: remove likewise**

Revisions in: Experiment 2: biomechanical measurement of cutaneous contact

The old sentence: Similar force-contact area relations were found in active touch as likewise found in passive touch.

[p.11 line 243] The new sentence: Similar force-contact area relations were found in active touch as found in passive touch.

10. **p. 13 line 220: "while the others were quite distinct from 1.48…" - do you mean averaged 1.48 cm^2?**

    We reported the averaged contact areas for the illusion case stimuli (10 kPa-4 mm, 90 kPa-6 mm, and 90 kPa-8 mm) and the distinct stimulus (10 kPa-8 mm) across all force levels. We changed the text to better clarify these results in Fig 5A and 5B.

Revisions in: Experiment 2: biomechanical measurement of cutaneous contact

The old sentence: In particular, data points for the three illusion cases were well clustered together across all force levels (0.90 ± 0.12 cm^2, mean ± SD), while the others were well separated (1.68 ± 0.18 cm^2).

[p.11 line 230] The new sentence: In particular, data points for the three illusion cases were well clustered across all force levels (average contact area: 0.90 ± 0.12 cm^2, mean ± SD), while the others were significantly distinct from them (average contact area: 1.68 ± 0.18 cm^2).

The old sentence: Specifically, data points for the illusion cases were clustered around 0.87 ± 0.10 cm^2 while the others were quite distinct from 1.48 ± 0.20 cm^2 across all force levels.

[p.11 line 247] The new sentence: Specifically, the average contact area for the three illusion cases is 0.87 ± 0.10 cm^2 while the other distinct stimulus derived an average contact area of 1.48 ± 0.20 cm^2 across all force levels.

11. **p. 13 line 226: cutaneous contact is indiscriminable – Experiment 2 focused on biomechanical measurements and so the term indiscriminable is not appropriate in this context. The results indicated that there was not a significant difference in the contact areas for the illusion-case stimuli.**

Yes, indeed. We have modified associated language throughout the manuscript.

Revisions in: Experiment 3: psychophysical evaluation of the elasticity-curvature illusion

The old sentence: The results of Experiment 2 support the hypothesis that cutaneous contact is indiscriminable between small-compliant and large-stiff spheres…

[p.12 line 256] The new sentence: The results of Experiment 2 support the hypothesis that cutaneous contact cues are not significantly different among illusion case spheres…

Revisions in: Experiment 2: biomechanical measurement of cutaneous contact

The old sentence: …we hypothesized that non-differentiable cutaneous contact might be observed between the small-compliant and large-stiff spheres.

[p.9 line 191] The new sentence: …we hypothesized that similar cutaneous contact cues might be observed among the illusion case spheres.

The old sentence: Because cues tied to contact area are identical, proprioceptive inputs evoked in active touch may be vital…

[p.11 line 252] The new sentence: Since cues tied to contact area are not significantly different, proprioceptive inputs evoked in active touch may be vital…

12. **p. 13 line 233: but their contact areas found non-differentiable – clumsy sentence, rewrite**

Revisions in: Experiment 3: psychophysical evaluation of the elasticity-curvature illusion

The old sentence: This illustrates that, when only cutaneous cues are available for discrimination but their contact areas found non-differentiable, these spheres indeed generate a perceptual illusion.

[p.12 line 275] The new sentence: These illustrate that when only cutaneous cues are available, but their contact areas do not differ, these spheres indeed are indiscriminable.

13. p. 13 p. 14 line 243: Can an illusion become discriminable? It is the stimuli being explored under certain conditions that makes them discriminable. (also line 257)

We agree and changed the text accordingly.

Revisions in: Experiment 3: psychophysical evaluation of the elasticity-curvature illusion

The old sentence: Then, to evaluate if the elasticity-curvature illusion becomes discriminable when adding proprioception to cutaneous contact…

[p.13 line 277] The new sentence: Then, to evaluate the discriminability of these stimuli when adding proprioception to cutaneous contact, …

The old sentence: Fourth, to validate the hypothesis that the proprioceptive cue of active finger displacement may help to discriminate the illusion, psychophysical evaluations were conducted in active touch.

[p.13 line 297] The new sentence: Fourth, to validate the hypothesis that the proprioceptive cue of active finger displacement may help to discriminate the illusion-case stimuli, psychophysical evaluations were conducted in active touch.

The old sentence: As illustrated in Fig 6A, the illusion was readily discriminable with a detection rate of 83.7% ± 6.9 (see S2 Table for detailed results).

[p.14 line 301] The new sentence: As illustrated in Fig 6A, the spheres were readily discriminable with an average correctness of 83.7% ± 6.9 (detailed in S3 Table) and an average sensitivity of 3.53 (detailed in S4 Table).

14. p. 15 line 263: Why is cutaneous contact illusory? (also line 284)

We agree with that “illusory” is not appropriate for the “cutaneous contact” and changed the text to correct this point.

Revisions in: Experiment 3: psychophysical evaluation of the elasticity-curvature illusion

The old sentence: …help in discriminating the stimuli amidst illusory cutaneous contact.

[p.14 line 306] The new sentence: …help in discriminating the stimuli amidst indiscriminable cutaneous contact areas.

Revisions in: A force-control movement strategy is optimal, efficient, and underlies softness perception

The old sentence: Amidst illusory cutaneous contact, participants’ volitionally control the contact forces they apply to soft objects.

[p.15 line 330] The new sentence: Amidst indiscriminable cutaneous contact cues, participants volitionally control the exploratory forces they apply to soft objects.

Revisions in: Abstract

The old sentence: …to optimally elicit and integrate proprioceptive inputs amidst illusory cutaneous contact.

[p.2 line 24] The new sentence: …to optimally elicit and integrate proprioceptive inputs amidst indiscriminable cutaneous contact.

15. p. 17 line 298: What does "overly grasped" mean?

Westling*, et al, 1984* found that applied grip force is balanced to optimize our motor behavior so that slipping between the skin and the object is prevented and the grip force does not reach exceedingly high values [2]. The "overly grasped" refers to the later condition: the contact pressure, derived from the applied grip force, reaches to a certain limit where the yield point is passed and plastic deformation occurs to the elastic object. We've changed this language below.

2. Westling G, Johansson RS. Factors influencing the force control during precision grip. Exp Brain Res. 1984;53: 277–284. doi:10.1007/BF00238156

Revisions in: A force-control movement strategy is optimal, efficient, and underlies softness perception

The old sentence: …the grip force within a safety margin, informed by skin mechanoreceptors, to prevent slipping or overly grasped [42,43].

[p.15 line 346] The new sentence: …the grip force within a safety margin, informed by skin mechanoreceptors, to prevent slipping or applying exceedingly high pressure [37,38].

16. p. 17 line 314: displacement to the – displacement in the

Done.

Revisions in: Discussion: Change of cutaneous contact as a cue to proprioception

[p.16 line 365] The new sentence: …induces a sensation of relative finger displacement in the stationary hand [20,46].

17. p.18 line 338: What is the evidence that this illusion "is naturalistic and commonplace in (not as) our daily lives?"

Our language here has been revised to reflect a recent paper we published regarding engineered stimuli as reasonable stand-ins for ecological fruits [3].

3. Xu C, He H, Hauser SC, Gerling GJ. Tactile exploration strategies with natural compliant objects elicit virtual stiffness cues. IEEE Trans Haptics. 2020;13: 4–10. doi:10.1109/TOH.2019.2959767

Revisions in: Discussion: A perceptual illusion inspired by everyday tasks

The old sentence: The attributes of elasticity and curvature are found in everyday, ecologically relevant tasks.

[p.17 line 383] The new sentence: The stimulus attributes of elasticity and curvature can be found in everyday, ecologically relevant tasks, e.g., judging the ripeness of a fruit for edibility.

[p.17 line 388] The new sentence: Herein, we address these issues by building spherical stimuli with covaried radii and elasticity which recapitulate important properties of ecologically compliant materials and mimic the contact profile of the skin surface's contacting elastic objects [9,10,22].

18. p. 19 line 364: phalange should be phalanx (singular form of phalanges)

Done.

Revisions in: Materials and methods: Geometry of the fingertip model

[p.18 line 415] The new sentence: Two simplified 2D finite element models were derived from the geometry of a 3D model of the human distal phalanx bone [31].

19. p. 24 line 468-469: It is not clear how randomization of stimuli has an effect on fatigue or inattention? There are various biases in participants' responses that can occur with fixed sequences of stimulus presentation which is why they are typically randomized in perceptual experiments.

We agree with the reviewer that a randomized design is optimal for discrimination tasks. In contrast, the fixed sequences of stimulus presentation would result in biases in participants' responses between trials. Specifically, when discriminating the stimulus pair via repeated stereotypical procedures, participants often tend to repeat their previous responses, and thus, the response biases would be carried over from one trail to another. For the same-different procedure used in this study, it has been often reported that sequences of binary response are not random [4]. Moreover, prior studies have shown that information gathered among sequential explorations are weighted unequally, due to fading memory representation [5,6].

In contrast, it is possible that a response-to-response dependency would be reduced if stimuli varied substantially and randomly [7]. Therefore, in order to reduce the biases and carry-over effects in participants' responses, we followed the experimental design mentioned in prior studies [6,8], and adapted it for use in our experimental tasks. We changed the text to better clarify this design.

4. Wagenaar WA. Sequential response bias in psychophysical experiments. Percept Psychophys 1968 35. 1968;3: 364–366. doi:10.1007/BF03396509
5. Metzger A, Drewing K. Switching between objects improves precision in haptic perception of softness. Lecture Notes in Computer Science (including subseries Lecture Notes in Artificial Intelligence and Lecture Notes in Bioinformatics). Springer Science and Business Media Deutschland GmbH; 2020. pp. 69–77. doi:10.1007/978-3-030-58147-3_8
6. Metzger A, Drewing K. Effects of stimulus exploration length and time on the integration of information in haptic softness discrimination. IEEE Trans Haptics. 2019;12: 451–460. doi:10.1109/TOH.2019.2899298

7. Verplanck WS, Blough DS. Randomized stimuli and the non-independence of successive responses at the visual threshold. J Gen Psychol. 1958;59: 263–272. doi:10.1080/00221309.1958.9710195
8. Jones LA, Tan HZ. Application of psychophysical techniques to haptic research. IEEE Trans Haptics. 2013;6: 268–284. doi:10.1109/TOH.2012.74

Revisions in: Experiment procedure

[p.24 line 543] The new sentence: Adapted from prior studies [51,52], the test order of discrimination trials was randomized to balance the carry-over effects in response bias [53].

[p.24 line 550] New sentences: Each stimulus pair was presented three times in a randomized order to balance the carry-over effects in sequential responses. There was a 15-seconds break between trials.

20. p. 25 lines 481-483: To be consistent with how the term is typically used in psychophysical experiments, the term “trial” should refer to the presentation of one stimulus pair which were presented successively separated by 2s. From each trial one response was provided by the participant. The time interval between trials was 15s.

Revisions in: Experiment 2: biomechanical measurement of cutaneous contact

The old sentence: By inspecting individual trials (Fig 5A), illusion case spheres (small-compliant and large-stiff) generated similar contact areas…

[p.10 line 226] The new sentence: By inspecting results from the example participant (Fig 5A), illusion case spheres (10 kPa-4 mm, 90 kPa-6 mm, and 90 kPa-8 mm) generated similar contact areas…

[p.11 line 240] New sentences: A sound alarm was triggered to end each exploration when the touch force reached the desired level. After each exploration, the ink-based procedure was conducted to measure the contact area between the finger pad and stimulus.

Revisions in: Measurement of contact area

The old sentence: This procedure was repeated until all trials were completed for the participant.

[p.22 line 488] The new sentence: This procedure was repeated until all measurements were completed for the participant.

Revisions in: Experiment procedure

The old sentence: For passive touch, all four stimuli were each indented into the finger pad at three force levels (1, 2, and 3 N) during three sessions respectively.

[p.23 line 510] The new sentence: For passive touch, all four stimuli were each indented into the finger pad at three force levels (1, 2, and 3 N) respectively.

[p.23 line 512] New sentences: There were three indentations for each stimulus at each indentation level per participant. All indentations were separated by a 20-second break. For active touch, the four stimuli were palpated by the index finger at three force levels which were behaviorally controlled. In particular, participants were instructed to actively press into the designated stimulus and a sound alarm was triggered to end the current exploration when their force reached the desired level. The ink-based procedure was used for each exploration. There were three explorations for each stimulus at each force level per participant. All explorations were separated by a 20-second break.

[p.24 line 532] New sentences: For passive touch, each trial consisted of discriminating one stimulus pair. Following the sampling order, spheres from the same pair were ramped into the fixed finger pad successively (Fig 4A).

[p.24 line 547] New sentences: Within each discrimination trial, a participant was instructed to explore compliance by palpating each of two spheres successively. When their touch force reached 2 N, a sound alarm was triggered to end that exploration. The interval between two explorations was set to 2-seconds as previously noted. Force and fingertip displacement were recorded simultaneously. Each stimulus pair was presented three times in a randomized order to balance the carry-over effects in sequential responses. There was a 15-seconds break between trials.

21. p. 25 line 488: remove some of the instances of "each"

Done.

Revisions in: Materials and methods: Data analysis

The old sentence: In particular, for each experimental task per each participant, all recordings of each tactile cue were normalized to the range of (0, 1) by sigmoidal membership function [5,26].

[p.25 line 559] The new sentence: In particular, for each experimental task, all recordings of each tactile cue were normalized to the range of (0, 1) by sigmoidal membership function [5,26].

The old sentence: After this transition, data from all participants were aggregated together for statistical analysis.

[p.25 line 561] The new sentence: After this transition was completed for each participant, all results were then aggregated together for statistical analysis.

Reviewer #2 Comments

General Comments:

The ms present a computational model of stimuli and finger pad from which a perceptual illusion is derived. The existence of the illusion is tested in a psychophysical experiment.

I very much like the approach to derive a potential perceptual illusion from the model, which sounds highly convincing to me. Also the first biomechanical test (Exp. 2) is nice and state-of-the art. In my view, however, the psychophysical experiment is confound because it does not carefully separate perceptual sensitivity from response bias. In addition the ms is not easy to read in places, in particular because important information is given too late. Also, implications of the results for decoupling cutaneous and proprioceptive cues are not well discussed. With new experimental data, however, the paper may definitely be worth to be published in a PLOS Comp BIOL.

> We thank the reviewer for the valuable and constructive comments. We agree in the need for further details in explaining the stimulus set, design of psychophysical experiments, and the statistical testing. We made revisions throughout the manuscript to address these points. Also, we revised the *Abstract*, *Introduction*, and *Discussion* to better articulate our findings. We prepared the following point-by-point responses to address the reviewer's comments.

Confound in Exp3:

A same/different task was used here, and the percentage of difference response was taken as a measure of perceptual performance. In this task, however, the percent "different" do not only depend on perceptual precision, but also on response tendencies (that is, the tendency to respond same or different in unclear cases). Typically, a control condition with two equal stimuli presented under equal conditions is included in order to dissociate perceptual precision from response tendency (cf. e.g. Green, D.M., Swets J.A. 1966, Signal Detection Theory and Psychophysics. New York: Wiley). In the present case, one control condition would have been required for each of the four conditions (passive with different force rates, active). However, as these control conditions were not included results may alternatively be explained by different response tendencies, e.g. by speculating that the less regular stimulations patterns in active vs.passive touch increase the percentage of "different" responses. I strongly recommend to redo experiment 3 including control conditions (and calculate perceptual precision separate from response tendency as described, e.g. in Green and Sweets, 1966) or, maybe better, to redo it using a response-bias free discrimination task (Which stimulus was softer?").

> The reviewer makes a good point. Indeed, given the "same-different" procedure, the derived perceptual sensitivity could be alternatively explained by response biases resulting from discriminating stimulus pairs. We had included a control condition in the experiment, but had not included those results in the manuscript. Therefore, we have now further analyzed this data and have provided more detailed information on the

employed stimulus set and the experimental design (including the control condition where two equal stimuli are presented under the same condition).

In our psychophysical experiments, we employed nine pairs of stimuli, including three control pairs where two equal stimuli are presented. As shown in S2 Table, by using an ordered sampling (with replacement) approach, we draw two stimuli from the three illusion case spheres to create a pair such that ordering matters and repetition is allowed. Thus, there are nine possibilities (stimulus pairs) and the ordering within each pair is determined.

S2 Table. Stimulus pairs drawn from the three illusion case spheres.

		The second stimulus		
		10 kPa-4 mm	90 kPa-6 mm	90 kPa-8 mm
The first stimulus	10 kPa-4 mm	(10,4) (10,4)	(10,4) (90,6)	(10,4) (90,8)
	90 kPa-6 mm	(90,6) (10,4)	(90,6) (90,6)	(90,6) (90,8)
	90 kPa-8 mm	(90,8) (10,4)	(90,8) (90,6)	(90,8) (90,8)

For each experimental condition (passive same/inverse/direct and active same force-rate), all nine stimulus pairs were presented and the trial order was randomized to balance the carry-over effects in response bias. By employing control pairs (two equal stimuli) and test pairs (two distinct stimuli), the perceptual precision could be better dissociated from response tendencies.

Our results show that, A) with respect to response biases, participants consistently exhibit better perceptual performance in discriminating control pairs (73.8% ± 6.0, compared with test pairs of 60.4% ± 30.0, mean ± SD, S3 Table), as would generally be expected. B) Discrimination performance on all stimulus pairs improved from “passive same” (46.1%) to “active same” (83.7%). This mainly results from an improved performance on test pairs since correctness for control pairs are relatively consistent across the four experimental conditions.

S3 Table. Results of psychophysical evaluations of all nine stimulus pairs.

Experimental Conditions	Discriminability (percentage of correctness)											
	10,4<br>10,4	10,4<br>90,6	10,4<br>90,8	90,6<br>10,4	90,6<br>90,6	90,6<br>90,8	90,8<br>10,4	90,8<br>90,6	90,8<br>90,8	Control Pairs	Test Pairs	**All Pairs**
**Passive same force-rate**	70.0	35.0	35.0	35.0	65.0	30.0	50.0	35.0	60.0	**65.0**	**36.7**	**46.1**
**Passive inverse force-rate**	85.0	55.0	50.0	40.0	80.0	40.0	40.0	25.0	60.0	**75.0**	**41.7**	**52.8**
**Passive direct force-rate**	65.0	75.0	70.0	80.0	80.0	65.0	75.0	90.0	90.0	**78.3**	**75.8**	**76.7**
**Active same force-rate**	70.0	66.7	90.0	83.3	73.3	100	86.7	96.7	86.7	**76.7**	**87.2**	**83.7**
**Average**	72.5	57.9	61.3	59.6	74.6	58.8	62.9	61.7	74.2	**73.8**	**60.4**	**64.8**

From the above, given that response bias doesn’t appear to be problematic, to further evaluate the discrimination results, we calculated the measure of sensitivity *d’*, which provides a bias-free measure that is not influenced by the observer’s response criterion

(S4 Table). For each stimulus pair condition, the sensitivity *d'* increases from ”passive same” to “active same force-rate.” This indicates that the participants become more sensitive to the difference within a stimulus pair, and perceptual performance is higher in active touch. Further details can be found in our responses to issue #5 in *Further points*.

S4 Table. Signal detectability of the three illusion case spheres.

		The signal detectability		
	Stimulus pair*	Hit rate	False-alarm rate	Sensitivity *d'*
**Passive same force-rate**	(10,4) & (90,6)	0.35	0.33	0.41
	(10,4) & (90,8)	0.43	0.35	0.84
	(90,6) & (90,8)	0.33	0.38	0.00
**Passive inverse force-rate**	(10,4) & (90,6)	0.48	0.18	1.81
	(10,4) & (90,8)	0.45	0.28	1.26
	(90,6) & (90,8)	0.33	0.3	0.50
**Passive direct force-rate**	(10,4) & (90,6)	0.78	0.28	2.61
	(10,4) & (90,8)	0.73	0.23	2.56
	(90,6) & (90,8)	0.78	0.15	3.13
**Active same force-rate**	(10,4) & (90,6)	0.75	0.28	2.47
	(10,4) & (90,8)	0.88	0.22	3.40
	(90,6) & (90,8)	0.98	0.20	4.72

* It includes all stimulus pairs that contain either of the two stimuli, e.g., pairs of [10,4-10,4], [10,4-90,6], [90,6-10,4], and [90,6-90,6] are all included in the column “(10,4) & (90,6)”.

Comprehensibility:

1. **Abstract: Is not easy to read, might focus more on what has actually done.**

   Please refer to our responses to the general comment from Reviewer #1, where extensive changes to the abstract were made.

2. **P3, 58: Please extend a bit on the conditions of the curvature illusion.**

   Please refer to our responses to issue #2 of Reviewer #1, where this was better clarified.

3. **P4, 62-63: The Doppler effect is a physical phenomenon, not a psychological one, and hence does not fit here. Please extend also a bit on the phenomenon reported in [15]**

   We agree the Doppler effect is not the best case and changed the text to fix this point.

Revisions in: Introduction

[p.4 line 60] New sentences: A further illusion, by analogy with the Aubert-Fleischl phenomenon in vision, indicates a possible misperception in the speed of a moving stimulus by touch [15]. Compared with tracking the stimulus with a guided hand movement (i.e., proprioception is available), observers can overestimate the stimulus speed with a stationary hand (i.e., tactile cues only).

4. **P4, 64-65: How do these phenomena shed light upon perceptuo-sensorimotor dependencies? Please extend. Please extend also a bit on applications.**

   We provided more details to better illustrate this point.

Revisions in: Introduction

[p.4 line 63] New sentences: These and other illusions shed light upon interdependencies of our sensorimotor and perceptual systems, i.e., processing mechanisms for the perception of object properties, e.g., size, orientation, and movement, are distinct from those underlying the mediation of those properties in sensorimotor control [16–19]. Furthermore, tactile illusions can serve as a tool in engineering applications where human perception could be manipulated, e.g., the stiffness of a virtual spring can provide a haptic sensation without a haptic display [9]. Meanwhile, illusions have also been considered as a metric to evaluate virtual environments by correlating the perceived realism with the illusion strength.

5. **P4, last para: The illusion needs to be better explained here.**

   We changed this paragraph to better explain the proposed illusion phenomenon.

Revisions in: Introduction

[p.5 line 82] The new paragraph: Here, we investigate a tactile illusion associated with softness perception, specifically, in exploring spherical stimuli with covaried elasticity and curvature. These physical attributes are routinely encountered, such as in judging the ripeness of spherical fruit. The illusion phenomenon is observed only in passive touch, when the finger is stationary and only non-distinct cutaneous cues are available for perception. The spheres, however, become readily discriminable when explored volitionally in active touch where finger proprioception is involved. The spheres therefore naturally dissociate relative contributions from cutaneous and proprioceptive cues in encoding softness, and shed light into how we volitionally explore compliant objects in everyday life.

6. **Fig. 2: How precisely is the location (x-axis) defined?**

   All the locations in Fig 2 only refer to positions of element nodes used in our finite element analysis. We employed in total 111 element nodes at the simulated epidermal-dermal interface to cover the locations (ranging from 0 to 15.2 mm). Therefore, there are approximately 7-8 nodes employed for each 1 mm. The modeling precision is around 0.1 mm/node. Note that the contact center (S4A Fig) was denoted as the origin and the x-axis directs to the fingertip. This is also illustrated in the figure below (Response to Reviewer 2 Figure 1).

Revisions in: Material and methods: Numerical simulations

[p.20 line 448] New sentences: …stress distributions at the epidermal-dermal interface (470 μm beneath the skin surface), calculated by averaging neighboring elements at

each interface node. Note that there were in total 111 element nodes employed to cover the locations from 0 to 15.2 mm.

Response to Reviewer 2 Figure 1: Distributions of the element nodes

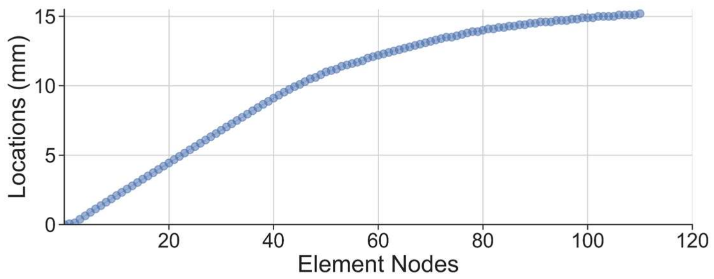

7. **Fig. 5A, B: Please indicate to what a single data point refers to (given there are several data points per condition)**

In Fig. 5A and B, each single data point represents the measured contact area from one single indentation at the corresponding force level (1, 2, or 3 N). The four stimuli were contacted by the finger with three repetitions per force level.

Revisions in: Fig 5. Caption.

[p.10 line 221] The new caption: …as opposed to the 10 kPa-8 mm sphere. Note that each data point represents the contact area measured from each indentation.

8. **P13, 219, 223: Which data entered the statistical tests and which and how many tests were used? Please indicate here.**

Regarding that same figure, we have added text at several points in the manuscript and include the details for each of the testing results. For Fig 5, the Mann–Whitney U test (α = 0.05, two-sided test) was applied to compare two data groups: a) all measurements for the 10 kPa-8 mm sphere; b) all measurements for the three illusion case spheres. The Cohen's d (the absolute value) was calculated for statistically significant results to evaluate the effect size.

Revisions in: Experiment 2: biomechanical measurement of cutaneous contact.

[p.10 line 227] New sentences: … illusion case spheres (10 kPa-4 mm, 90 kPa-6 mm, and 90 kPa-8 mm) generated similar contact areas while the distinct sphere (10 kPa-8 mm) afforded higher contact areas. There was a significant difference between contact areas of the illusion case and distinct spheres across all force levels ($U = 0.0$, $p < 0.0001$, $d = 4.81$).

[p.11 line 235] New sentences: …the trace for the 10 kPa-8 mm sphere was distinct. Specifically, there was a significant difference between contact areas of the illusion case and distinct spheres across all force levels ($U = 87.0$, $p < 0.0001$, $d = 4.49$).

[p.11 line 245] New sentences: …while the 10 kPa-8 mm sphere exhibited higher values. There was a significant difference between results of the illusion case and distinct spheres across all force levels (U = 0.0, p < 0.0001, *d* = 3.73).

[p.11 line 250] New sentences: …more distinct relationship. Specifically, there was a significant difference between the contact areas of the illusion case and distinct spheres across all force levels (U = 0.0, p < 0.0001, *d* = 4.49).

9. **P13, 232: What exactly was the task/instruction of participants? Why is the result called a "detection rate" rather than a percentage of correct discriminations?**

   The same-different procedure was employed herein. With this procedure, each trial consists of explorations of two stimuli that are either the same or different (i.e., including control and test pairs). The participant's task is to explore the stimuli sequentially and answer the question - "whether the compliances of the two stimuli are the same or different". The answer should be either "same" or "different". We agree the "percentage of correct discriminations" fits better and changed the text accordingly.

Revisions in: Experiment 3: psychophysical evaluation of the elasticity-curvature illusion

[p.12 line 271] The new sentence: …where indentation rate was controlled at 1 N/s (Fig 6A), participants were not able to discriminate the stimuli (average percentage of correct discriminations: 46.1% ± 5.7).

[p.13 line 280] The new sentence: …at a higher force-rate (2 N/s) than the harder stimulus (0.5 N/s), participants were still unable to discriminate the compliances with an average percentage of correctness of 52.8% ± 6.7.

[p.14 line 302] The new sentence: As illustrated in Fig 6A, the spheres were readily discriminable with an average correctness of 83.7% ± 6.9 (detailed in S3 Table) and an average sensitivity of 3.53 (detailed in S4 Table).

10. **P14, 246: What is exactly meant by a "higher force-rate" (over time)? And how much higher was it? Overall, the rationale of the force-rate manipulation is unclear at this point of the ms and should be better explained. It is also unclear how the active condition (Fig. 6A) can be a "same-force rate" condition**

    As shown below in Response to Reviewer 2 Table 1 and Figure 2, we controlled the indentation force-rate at 0.5, 1, and 2 N/s for the "lower", "same", and "higher force-rate" control mode and different experimental tasks respectively.

Response to Reviewer 2 Table 1: Force-rate control for different experimental conditions

		Force-rate (N/s)	
		(10, 4)	(90, 6) & (90, 8)
Experimental Conditions	Passive same force-rate	1.0	1.0
	Passive inverse force-rate	2.0	0.5

	Passive direct force-rate	0.5	2.0

Response to Reviewer 2 Figure 2: The triangle-wave indentation force profile

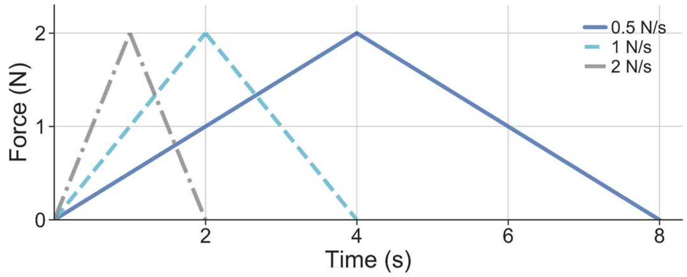

Rationale of the force-rate manipulation:

A force-control strategy is important from a number of perspectives. First, a force-manipulation contact is essential to compensate for the remodeling of the skin over time [9]. Second, we prioritize the applied force to achieve high level performance [10–12]. Furthermore, our prior work demonstrated that force-rate cues correlate with the object softness. In active touch, significantly higher force-rate was applied to the harder stimuli, as compared to the softer ones [3,13]. This “direct relationship” between force-rate and softness was also found in passive touch [14,15]. However, when force-rate was manipulated to be contradicted with the softness, our discriminability could be impacted [15]. In order to further investigated this force-rate control mechanism and its correlation with elicited cutaneous cues, we employed different force-rate control conditions herein.

Response to Reviewer 2 Figure 3: The goodness of fit for the force-rate cue, across the 10 participants.

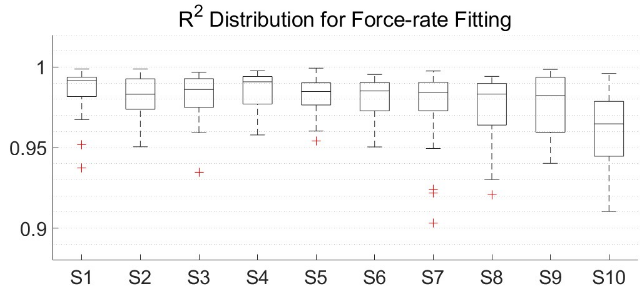

The same force-rate in active touch:

As shown in Fig 6B, there is no significant difference among the force-rate values for the three illusion case spheres. Considering the terminal force level was constrained as 2 N, these results indicated that participants volitionally generate non-distinct force cues for discrimination. Therefore, we denoted this as the “active same force-rate” condition.

We added a new section to clarify how to obtain the force-rate value. A linear regression was applied to the force ramp and the derived slope was noted as the force-rate. The goodness of fit was shown in Response to Reviewer Figure 3. The average R-square was 0.98 ± 0.02 across all participants.

Revisions in: Experiment 3: psychophysical evaluation of the elasticity-curvature illusion

[p.12 line 268] New sentences: Participants (n = 10) were first instructed to discriminate the illusion case spheres in passive touch. Meanwhile, to further investigate the utility of temporal cues in augmenting our discrimination performance, the indentation force-rate was systematically modulated in three different experimental conditions.

[p.12 line 271] The new sentence: In the “passive same force-rate” task, where indentation rate was controlled at 1 N/s (Fig 6A), …

[p.13 line 279] The new sentence: …where the softer stimulus was indented “inversely” at a higher force-rate (2 N/s) than the harder stimulus (0.5 N/s), …

[p.13 line 287] The new sentence: …where the softer stimulus was indented “directly” at a lower force-rate (0.5 N/s) than the harder stimulus (2 N/s), …

[p.13 line 299] New sentences: …under fully active, volitional sensorimotor control. Non-distinct force-rate cues were applied in exploring the illusion case spheres (Fig 6B), therefore, this experimental condition was denoted as “active same force-rate”.

[p.14 line 310] The new sentence: Specifically, given the same terminal indentation force level (2 N) and non-distinct force-rate cues (no significantly difference detected) among illusion cases, significantly higher displacement was applied for the softer spheres (10 kPa-4 mm vs. 90 kPa-6 mm: U = 8786.0, $p < 0.0001$, $d = 0.74$; 10 kPa-4 mm vs. 90 kPa-8 mm: U = 5737.5, $p < 0.0001$, $d = 1.18$).

The new section in Material and methods: Measurement of force and displacement

[p.22 line 495] The gross contact readings from the force and laser sensor were smoothed to remove electrical artifacts by a moving filter with a window of 100 neighboring readings. The ramp segments of the force curves were then extracted based on first-order derivatives [5]. A linear regression was applied to the segments and the derived slope was noted as the force-rate. On the other hand, the fingertip displacement was calculated as the absolute difference between the initiation and conclusion of each movement.

11. P14, 248: Which data entered the statistical test and which test was used? Please indicate here.

In Fig 6A, for each of the four experimental conditions, one data point represents the overall percentage of correct discriminations for one participant. This was also noted in Fig 6. Caption. All data points for the same experimental conditions were included into one group. The Mann–Whitney U test ($\alpha = 0.05$, two-sided test) was applied to compare any two data groups (across experimental conditions) and the Cohen’s d (the absolute value) was calculated to evaluate the effect size.

Revisions in: Experiment 3: psychophysical evaluation of the elasticity-curvature illusion

[p.13 line 281] The new sentence: However, this discrimination correctness (with all participants aggregated) was significantly higher compared with the "passive same force-rate" condition ($U = 24.0$, $p < 0.05$, $d = 1.03$)

[p.13 line 289] The new sentence: This result of correctness (with all participants aggregated) was significantly higher compared to the "passive inverse force-rate" task ($U = 0.5$, $p < 0.0001$, $d = 3.72$) and the "passive same force-rate" task ($U = 0.0$, $p < 0.0001$, $d = 4.33$).

[p.14 line 303] The new sentence: This presents significantly higher discrimination correctness (with all participants aggregated) compared to the "passive direct force-rate" task ($U = 21.0$, $p < 0.05$, $d = 1.08$).

12. **Exp. 3: How was it exactly achieved that the finger was not differently displaced in the passive conditions, and how successful were these measures? Please report in the main ms.**

We employed several physical measures to constrain the forearm and index finger. First, the participant's forearm was supported by a stationary armrest, which was bolted onto the base of the motion stage. The forearm could also be constrained by Velcro straps when there was not sufficient friction. Second, we used a semicircular fixture to steadily hold the index finger. This fixture was made from the PVC pipe and was bolted onto the surface of the armrest. The inner diameter was customized based on the dimensions of each participant's distal phalanx to eliminate any shear movement. Last, we've considered to use Velcro straps to further tight the index finger with the fixture but this measure would inevitably pose pressure onto the finger and cause hyperemia. In our pilot trails, this measure was reported as discomfortable and would further deter perceptual performance. Therefore, this measure was finally not employed in this work.

Revisions in: Material and methods: Stimuli and experimental apparatus

[p.21 line 469] New sentences: Physical measures were employed to eliminate any movement of the finger pad during the indentation. First, the participant's forearm was supported by a stationary armrest bolted onto the base of the motion stage. Velcro straps were further used to constrain if any slipperiness was detected. Second, a plastic semicircular fixture was installed to hold the index finger. The inner diameter was determined based on the dimensions of participants' distal phalanx to fasten the distal and proximal interphalangeal joints. Finally, the finger pad was held at approximately 30 degrees relative to the stimulus surface.

13. **P21, 407, and P23, 444: How many stimuli were made out of silicone? Only four or the nine from the simulations? Which were used?**

A) In the finite element analysis, nine stimuli (3 radii by 3 elasticity) were created for simulation, with three moduli (10, 50, 90 kPa) and three radii (4, 6, and 8 mm). B) All these nine stimuli were then fabricated using silicone-elastomer. C) Four stimuli (illusion case: 10 kPa-4 mm, 90 kPa-6 mm, and 90 kPa-8 mm; distinct case: 10 kPa-8 mm) were

used in Experiment 2 (biomechanical experiment). The three illusion case stimuli were used in Experiment 3 (psychophysical experiment).

Revisions in: Material and methods: Stimuli and experimental apparatus

[p.20 line 459] The new sentence: Nine compliant stimuli (3 radii by 3 elasticity) were constructed from a room temperature curing silicone elastomer (BJB Enterprises, Tustin, CA; TC-5005 A/B/C).

Revisions in: Material and methods: Experiment procedure

[p.23 line 508] The new sentence: …both passive and active touch with four stimuli (illusion case: 10 kPa-4 mm, 90 kPa-6 mm, and 90 kPa-8 mm; distinct case: 10 kPa-8 mm).

[p.23 line 521] The new sentence: …active touch with the three illusion case stimuli.

Revisions in: Results: Experiment 2: biomechanical measurement of cutaneous contact

[p.10 line 212] The new sentence: Each of the four spheres (three illusion case stimuli and one distinct stimulus) was indented into the finger pad with a triangle-wave force profile...

14. P24, 465-477: How was the order of trials from different (passive) conditions? Were they blocked or random? How was the order of stimuli in each trial?

Within each experimental condition, i.e., "passive same/inverse/direct" and "active same" force-rate, all trials were randomized to balance the carry-over effects in response biases. Trials from the same experimental condition were grouped together and conducted within one block. Test order of the four experimental conditions (blocks) were randomized for each participant. For information about stimulus ordering, please refer to our responses to issue "*Confound in Exp3*". Detailed information was shown in S2 Table.

Revisions in: Material and methods: Experiment procedure

[p.24 line 532] The new section: For passive touch, each trial consisted of discriminating one stimulus pair. Following the sampling order, …In the "passive inverse force-rate" task, higher force-rate was applied for the soft stimulus while the lower force-rate was applied for the hard stimulus, …In the "passive direct force-rate" task, force-rate was applied in a direct positive relation with the stimulus modulus. The 10 kPa-4 mm sphere was indented at 0.5 N/s to 2 N and the two 90 kPa spheres were indented at 2 N/s to 2 N. For each experimental task, each of the nine stimulus pairs was presented twice. Adapted from prior studies [51,52], the test order of discrimination trials was randomized to balance the carry-over effects in response bias [53].

[p.24 line 552] New sentences: Note that trials under the same experimental task were grouped together and conducted within one block. Test order of the four experimental tasks (blocks) were randomized for each participant.

Further points:

1. **Fig. 2, Fig. 3: it is very nice to see similarity and dissimilarity of stress distributions and other measures here. I wonder whether in addition an aggregate number of (dis-)similarities was available that should then be reported for all 9 stimuli.**

   We did further analysis on the three attributes and added new figure/text to clarify. In order to have an aggregated number to quantify the similarity, we calculated the average responses at each force load. All element nodes within the range of [0, 6] mm were included for analysis. We selected this range since it is the midpoint of the spheres' radii (4, 6, and 8 mm) and it captures the region of interest for contact simulation.

The new S3 Fig

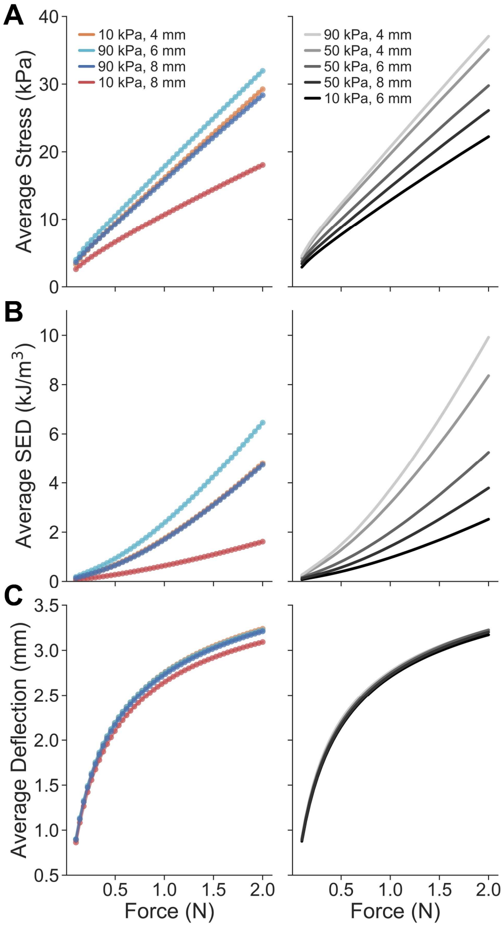

Revisions in: Supporting information: Perceptual cues predicted in the computational modeling

The new figure caption: **S3 Fig. Average skin mechanics responses from the computational model over the same contact region for cutaneous cues.** For the intermediate force loads, average responses were quantified over the same contact region for tactile cues of (A) stress, (B) SED, and (C) surface deflection. The average stress/strain distributions overlap for the illusion case spheres, while similar average deflection cues were derived from all nine stimuli.

[SI p.6 line 55] New sections: Based on the analysis of stress/strain distributions and surface deflection, average responses of these contact cues were calculated to further quantify the similarity/difference among the illusion case spheres (10 kPa-4 mm, 90 kPa-6 mm, and 90 kPa-8 mm), the distinct sphere (10 kPa-8 mm), and all the others. In particular, for all element nodes within 6 mm at the contact interface, i.e., the location range is [0, 6] mm, the mean response of each contact cue was calculated at each force load (S3 Fig).

[SI p.6 line 60] Compared with the other six spheres, we found that the illusion case spheres generate overlapped stress/strain responses. However, similar surface deflection results were observed upon contact for all nine stimuli. Specifically, compared with the distinct sphere (10 kPa-8 mm), overlapping stress/strain-force curves were obtained for the three illusion case spheres (S3 Fig). The average stress distributed over the contact region was 17.15 ± 7.70 kPa (mean ± SD) for three illusion case spheres across all force loads, as compared to the distinct sphere of 10.88 ± 4.48 kPa. Likewise, the average SED was 2.31 ± 1.63 kJ/m3 for the illusion case spheres across all force loads, as compared to the distinct sphere of 0.74 ± 0.47 kJ/m3. Last, the average surface deflection across force loads was 2.59 ± 0.59 mm for the illusion case spheres, as compared to the distinct sphere of 2.49 ± 0.57 mm.

Revisions in: Results: Experiment 1: computational modeling of the elasticity-curvature illusion

[p.8 line 160] New sentences: In addition, average cutaneous responses over the contact region were also similar between the three spheres (S3 Fig). These results demonstrate that small-compliant and large-stiff stimuli can generate nearly identical cutaneous cues, and therefore, naturally dissociate from proprioceptive movements.

2. **P16, Discussion: please explain in greater detail how you conclude from your data on the strategy of volitional force control.**

We changed the text in *Discussion* to clarify. In active discrimination task, the terminal exploratory force was behaviorally controlled to be 2 N. Derived from the calculation of force-rate for each indentation, we found that the rate change of touch force was also volitionally controlled to be non-distinct among the three illusion case spheres (Fig 6B). Participants instead moved their fingers to elicit significantly different displacements for discrimination (Fig 6C). Given the same terminal touch force and force-rate cue, we concluded that participants volitionally applied consistent force trajectories between stimuli and thereby utilize the resultant distinct fingertip displacements for discrimination.

Revisions in: Discussion: A force-control movement strategy is optimal, efficient, and underlies softness perception

[p.15 line 330] New sentences: Amidst indiscriminable cutaneous contact cues, participants volitionally control the exploratory forces they apply to soft objects. Specifically, the terminal indentation force, as well as the rate change of touch force was volitionally controlled to be non-distinct among the illusion case spheres (Fig 6B).

Indeed, participants actively move their fingers to apply consistent force trajectories and thereby evoke significantly differences in fingertip displacement cues for softness discrimination. These fingertip displacements are proprioceptive by nature and critical to the discrimination of the illusion case stimuli (Fig 6C).

3. **P17, 313-314, "… skin deformation of this kind naturally includes a sensation of relative finger displacement … ": This effect should occur for both the passive direct force-rate, and the passive indented force-rate condition, and hence cannot separately explain the behaviour in the passive direct force-rate condition, as done here.**

We changed the text to clarify this effect. When force-rate cue was made available in "passive inverse/direct force-rate" task, discrimination correctness indeed improved from "passive same force-rate" task. The force-rate cue indeed elicited changes in gross contact areas (S5 Fig). Prior studies have demonstrated that the change of contact area could induce a sensation of finger displacement. Thus, force-rate cue could be perceived as an alternative cue, and the improved performance (passive inverse/direct) could be derived from proprioceptive sensation of this kind [16–19].

Furthermore, discrimination performance also improved from "passive inverse" to "passive direct" force-rate task. Indeed, this "direct relationship" between force-rate and softness has been found both in passive touch [14,15], and fully active discrimination task, where significantly higher force-rate was consistently applied to the harder stimuli, as compared to the softer stimuli [3,13]. Therefore, besides the alternative proprioceptive input, the direct mapping between force-rate and softness could also help discrimination.

Revisions in: Discussion: Change of cutaneous contact as a cue to proprioception

[p.16 line 350] The new sentence: In passive touch, we observe that participants can discriminate the illusion case stimuli, particularly in the "passive direct force-rate" case, with a correctness of about 77% (Fig 6A).

[p.16 line 354] The new sentence: We hypothesize that the modulation of force under "passive direct/inverse force-rate" condition – where the softer stimulus was indented "directly/inversely" at a lower/higher force-rate than the harder stimulus – provides an alternate perceptible input during the dynamic contact phase, also tied to finger proprioception.

[p.16 line 368] The new sentence: Therefore, when passively exploring the illusion case spheres under the modulation of force-rate, the improved discriminability is likely derived from the proprioceptive sensation elicited by the change of contact area, which is originally induced by the force-rate cue.

4. **P24, 456-457, "nine stimulus pairs were drawn from the three illusion cases": Please explain in greater detail. Make clear what the three cases are and how nine pairs can be drawn from three cases.**

Please refer to our responses to issue “Confound in Exp 3”. We changed the text and presented the new S2 Table to clarify this point.

Revisions in: Material and methods: Experiment procedure

[p.23 line 520] New sentences: <u>In Experiment 3, psychophysical discrimination experiments were conducted for both passive and active touch with the three illusion case stimuli. Following the rule of ordered sampling with replacement, nine stimulus pairs were drawn from the three illusion case spheres and were prepared for psychophysical evaluation. The stimulus ordering within each pair was determined by the sampling results (see S2 Table for detailed assignments).</u>

5. **P24, 465-477, from these lines and table S2 in the supplement I concluded that the three cases were 10 kPa-4mm with 90kPA 8mm, 10 kPa-4mm with 90kPA 6mm, and 90 kPa-6mm with 90kPA 8mm. In the latter pair, compliances of the two stimuli were the same. Was the response recoded in that case? That is was a “same”-response for that pair considered a “correct” response in fig. 6 A? In table S2 it appears that this was (incorrectly) not the case, given that in the passive same force ratio condition the percentage for the latter pair was as low as for the other two pairs. Please clarify.**

We made changes throughout the manuscript to explicitly clarify the confusion. **First**, the three illusion case spheres were: 10 kPa-4 mm, 90 kPa-6 mm, and 90 kPa-8 mm. **Second**, nine stimulus pairs were drawn for Exp 3. Please refer to our responses to issue “Confound in Exp 3” and S2 Table for details on the stimulus pairs. **Third**, we used the same-different procedure in Exp 3. For details on the ordering and task, please refer to our responses to issue “Confound in Exp 3”, issue #9 and #14. **Fourth**, we changed S3 Table to clarify any confusion. The percentage of correctness for each stimulus pair was derived with all participants aggregated. Aggregated results were also derived for control pairs (with two equal stimuli), test pairs (with two distinct stimuli), and all pairs.

Furthermore, with respect to the pair of (90,6 – 90,8) and (90,8 – 90,6), the correct response is “same” and was recorded in Fig 6A. As in S3 Table, results for these two pairs were mostly consistent with others. Align with our prior findings [13], these two pairs were indeed challenging. Since these two stimuli only vary with the curvature, not the compliance, the psychophysical question would inevitably pose certain presupposition and decouple compliance perception with the curvature.

Finally, in S4 Table, sensitivity for the (90,6) and (90,8) stimuli become higher from ”passive same” to “active same” experimental condition. This indicates an improved discriminability. Note that we used the differencing rule assumption to derive *d’* and these values are finally determined from Table A 5.4 in [20]. Please refer to our responses to issue “Confound in Exp 3” for detailed results.

Revisions in: Experiment 3: psychophysical evaluation of the elasticity-curvature illusion

[p.12 line 273] New sentences: In addition, the sensitivity measure *d'* was also calculated under the assumption of differencing rule [39]. Average *d'* of 0.42 yielded a chance performance across all stimulus pairs (detailed in S4 Table).

[p.13 line 283] The new sentence: Average *d'* value of 1.19 across stimulus pairs also indicated an improved, but still poor discrimination sensitivity under this condition (detailed in S4 Table).

[p.13 line 292] The new sentence: The values of participants' sensitivity were also improved for all stimulus pairs (detailed in S4 Table).

[p.14 line 301] The new sentence: As illustrated in Fig 6A, the spheres were readily discriminable with an average correctness of 83.7% ± 6.9 (detailed in S3 Table) and an average sensitivity of 3.53 (detailed in S4 Table).

References

3. Xu C, He H, Hauser SC, Gerling GJ. Tactile exploration strategies with natural compliant objects elicit virtual stiffness cues. IEEE Trans Haptics. 2020;13: 4–10. doi:10.1109/TOH.2019.2959767
9. Wang Y, Baba Y, Lumpkin EA, Gerling GJ. Computational modeling indicates that surface pressure can be reliably conveyed to tactile receptors even amidst changes in skin mechanics. J Neurophysiol. 2016;116: 218–228. doi:10.1152/jn.00624.2015
10. Kaim L, Drewing K. Exploratory strategies in haptic softness discrimination are tuned to achieve high levels of task performance. IEEE Trans Haptics. 2011;4: 242–252. doi:10.1109/TOH.2011.19
11. Tan HZ, Durlach NI, Beauregard GL, Srinivasan MA. Manual discrimination of compliance using active pinch grasp: The roles of force and work cues. Percept Psychophys. 1995;57: 495–510. doi:10.3758/BF03213075
12. Zoeller AC, Drewing K. A Systematic Comparison of Perceptual Performance in Softness Discrimination with Different Fingers. Attention, Perception, Psychophys. 2020; 1–14. doi:10.3758/s13414-020-02100-4
13. Xu C, Hauser SC, Wang Y, Gerling GJ. Roles of force cues and proprioceptive joint angles in active exploration of compliant objects. 2019 IEEE World Haptics Conference (WHC). IEEE; 2019. pp. 353–358. doi:10.1109/WHC.2019.8816159
14. Li B, Hauser S, Gerling GJ. Identifying 3-D spatiotemporal skin deformation cues evoked in interacting with compliant elastic surfaces. IEEE Haptics Symposium, HAPTICS. IEEE Computer Society; 2020. pp. 35–40. doi:10.1109/HAPTICS45997.2020.ras.HAP20.22.5a9b38d8
15. Hauser SC, Gerling GJ. Force-rate cues reduce object deformation necessary to discriminate compliances harder than the skin. IEEE Trans Haptics. 2018;11: 232–240. doi:10.1109/TOH.2017.2715845
16. Moscatelli A, Bianchi M, Serio A, Terekhov A, Hayward V, Ernst MO, et al. The change in fingertip contact area as a novel proprioceptive cue. Curr Biol. 2016;26: 1159–1163. doi:10.1016/j.cub.2016.02.052
17. Proske U, Gandevia SC. The proprioceptive senses: Their roles in signaling body shape, body position and movement, and muscle force. Physiol Rev. 2012;92: 1651–1697. doi:10.1152/physrev.00048.2011
18. Edin BB, Johansson N. Skin strain patterns provide kinaesthetic information to the human central nervous system. J Physiol. 1995;487: 243–251. doi:10.1113/jphysiol.1995.sp020875
19. Moscatelli A, Bianchi M, Serio A, Al Atassi O, Fani S, Terekhov A, et al. A change in the fingertip contact area induces an illusory displacement of the finger. Lecture Notes in Computer Science (including subseries Lecture Notes in Artificial Intelligence and Lecture Notes in Bioinformatics). Springer Verlag; 2014. pp. 72–79. doi:10.1007/978-3-662-44196-1_10
20. Macmillan NA, Creelman CD. Detection theory: A user's guide. Cambridge University Press; 1991.

Reviewer #3 Comments

General Comments:

This study investigates the sensory cues involved in the perception of softness. Specifically, using computational modelling, a novel illusion is identified, whereby objects that differ in elasticity will feel the same when indented passively into the skin, but will feel different when touched actively using comparable forces. The illusion works by employing objects of different sizes such that their tactile imprint onto the skin is identical, while additional proprioceptive input in the active condition will disambiguate between the objects.

The paper is well-written and easy to follow. The study itself is cleverly designed and it is especially great to see the interplay between computational modelling and psychophysical measurements used to establish a novel illusion. My comments mainly focus on clarity and context.

We thank the reviewer for the constructive and valuable suggestions. The reviewer brings up several points concerning the computational modeling, as well as the details in psychophysical experiments. We attempt to clarify.

1. **The study focuses exclusively on the static part once the force has reached its plateau. However, many tactile afferents respond exclusively to the dynamic period and the overall population firing rate might well be higher in the initial contact phase than in the plateau phase. Would the skin mechanics model predict that the illusion should also be apparent during this phase? Or would contact differ instead, but for some reason this difference might not translate into a perceptual effect? Further detail is needed here and this problem should be discussed and ideally addressed with further analysis.**

Indeed, the contact mechanics may impact afferent responses during dynamic contact. To address this, we re-simulated the contact ramp phase. Results indicate that the illusion case spheres may still afford non-distinct cutaneous cues during the stimulus ramp, and thus, could elicit similar afferent responses from both slowly and rapidly adapting mechanoreceptors and may result in an illusory experience.

Specifically, by inserting 50 more discretized time points into the simulation procedure, we interpolated the force load to re-simulate the stimulus ramp phase. As in S7 Fig, illusion case spheres could indeed generate overlapping stress distributions across all intermediate force loads. Over the contact time-course, average cutaneous cues were consistently overlapped (please see S3 Fig and refer to our responses to issue “Further points” #1 from Reviewer #2). Furthermore, the rate of change of these responses were non-distinct, especially for (10,4) and (90,8) spheres (S8 Fig). These modeling observations indicate that, throughout the whole contact time-course, similar afferent responses from slowly/rapid adapting mechanoreceptors might be elicited by the illusion case spheres, and thus, may render an illusory experience in discriminating the compliances.

The new S7 and S8 Fig:

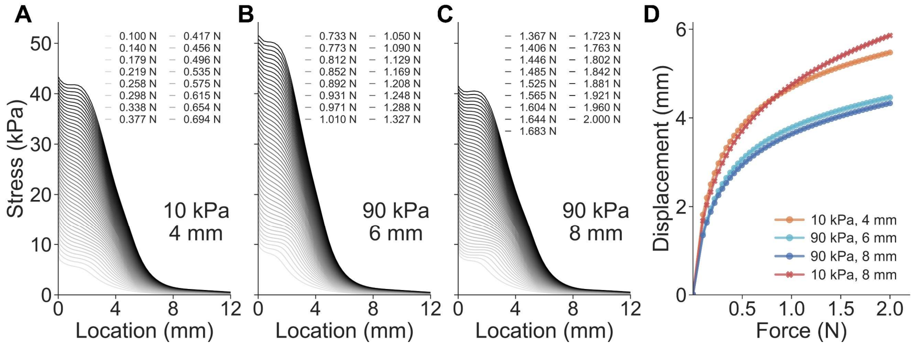

The new figure caption: **S7 Fig. Cutaneous and proprioceptive responses simulated during dynamic contact.** Stress distributions at contact locations for the three illusion case spheres: (A) 10 kPa-4 mm, (B) 90 kPa-6 mm, and (C) 90 kPa-8 mm. (D) Proprioceptive cues of finger displacement are simulated for all discretized force load during the ramp phase.

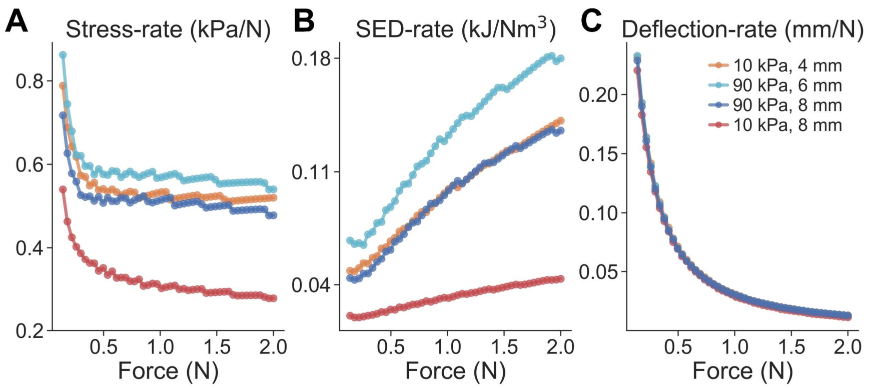

The new figure caption: **S8 Fig. The rate of change in cutaneous responses during dynamic contact.** Derived from S3 Fig, the rate of change of averaged (A) stress, (B) SED, and (C) surface deflection are calculated for the contact ramp phase. Note that within the simulation procedure, time points are linearly coupled with force loads, i.e., 0.5 N is applied at 0.25 sec and 1.5 N is applied at 0.75 sec, etc.

Revisions in: Experiment 1: computational modeling of the elasticity-curvature illusion

[p.9 line 182] The new section: Besides analyzing response variables only at the steady-state, the stimulus-ramp phase was further simulated to evaluate how contact mechanics would derive responses during the dynamic contact (detailed in Supporting information). Overall, the illusion case spheres could still afford nearly identical cutaneous responses during the stimulus ramp (S3 and 7 Fig). The rate of change in stress distributions, SED, and surface deflection cues consistently overlap (S8 Fig). This indicates that, throughout contact time-course done in silico, similar afferent responses from both slowly and rapidly adapting mechanoreceptors might be elicited among the illusion case spheres, and thus, may render an illusory experience in discriminating their compliances.

Revisions in: Supporting information

[SI p.6 line 55] The new section: Based on the analysis of stress/strain distributions and surface deflection, average responses of these contact cues were calculated to further quantify the similarity/difference among the illusion case spheres (10 kPa-4 mm, 90 kPa-6 mm, and 90 kPa-8 mm), the distinct sphere (10 kPa-8 mm), and all the others. In particular, for all element nodes within 6 mm at the contact interface, i.e., the location range is [0, 6] mm, the mean response of each contact cue was calculated at each force load (S3 Fig).

[SI p.6 line 60] The new section: Compared with the other six spheres, we found that the illusion case spheres generate overlapped stress/strain responses. However, similar surface deflection results were observed upon contact for all nine stimuli. Specifically, compared with the distinct sphere (10 kPa-8 mm), overlapping stress/strain-force curves were obtained for the three illusion case spheres (S3 Fig). The average stress distributed over the contact region was 17.15 ± 7.70 kPa (mean ± SD) for three illusion case spheres across all force loads, as compared to the distinct sphere of 10.88 ± 4.48 kPa. Likewise, the average SED was 2.31 ± 1.63 kJ/m^3 for the illusion case spheres across all force loads, as compared to the distinct sphere of 0.74 ± 0.47 kJ/m^3. Last, the average surface deflection across force loads was 2.59 ± 0.59 mm for the illusion case spheres, as compared to the distinct sphere of 2.49 ± 0.57 mm.

[SI p.12 line 150] To further investigate how contact with the illusion case spheres might impact afferent responses during the dynamic contact phase, especially for rapidly adapting mechanoreceptors, we simulated the ramp phase by interpolating loads and derived response variables with higher precision. In particular, we evenly inserted 48 more time points in the simulation procedure and thus, interpolated the force load of 0 to 2 N with total 50 discretized intermediate loads to fully simulate dynamic contact. These discretized loads are shown in S7 Fig as legends. Note that the first load (0 N) is omitted.

[SI p.13 line 161] As illustrated in S7A-C Fig, across all intermediate force loads, the illusion case spheres (i.e., 10 kPa-4 mm, 90 kPa-6 mm, and 90 kPa-8 mm) indeed generated similar stress distributions at contact locations. Specifically, stress curves of the 10 kPa-4 mm and 90 kPa-8 mm nearly overlapped. Based on these model predictions, these results indicate that, during the dynamic contact phase, i.e., early ramp stage, afferent responses that might be elicited given a stress input would be expected to be nearly consistent among the illusion case spheres.

[SI p.13 line 172] To quantify the similarity of cutaneous cues from illusion case spheres, averaged responses (stress/SED/surface deflection) over a contact region were calculated (S3 Fig). Compared with the other six spheres, we found that illusion case spheres generate overlapped stress/strain/deflection responses over the whole contact time-course. Furthermore, the rate of change of these cutaneous cues consistently overlap (S8 Fig). These indicate that upon dynamic contact the illusion case spheres might be expected to elicit similar afferent responses from rapidly adapting

mechanoreceptors. Therefore, it is likely that compliances of the illusion case spheres are consistently indiscriminable during the stimulus ramp.

2. **Figure 1: It would help here to explicitly show the dermis/epidermis border in the illustration to indicate where stress mostly impacts neural responses.**

We modified the figure and the text to better illustrate this point. As shown in the new Fig 1, the epidermis consisted of two layers of element nodes and the dermis consisted of three layers of nodes. Stress distributions and strain energy density at the epidermal-dermal interface (470 μm beneath the skin's surface), where Merkel cell end-organs of slowly adapting type I afferents and Meissner corpuscles of rapidly adapting afferents reside, were calculated as cutaneous cues derived from contact interactions.

The new Fig 1:

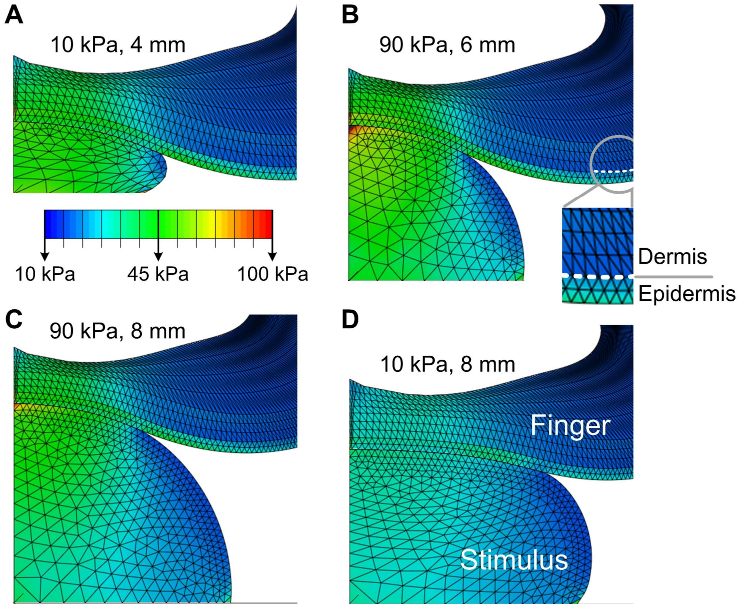

Revisions in: Fig 1. Caption

[p.7 line 132] The new caption: **Computational modeling of contact mechanics with compliant spheres.** Spatial distributions of stress are simulated at a load of 2 N for contact with spheres of (A)10 kPa-4 mm, (B) 90 kPa-6 mm, (C) 90 kPa-8 mm, and (D) 10 kPa-8 mm respectively. The epidermal-dermal interface was indicated in (B) and was consistently modeled for all simulation conditions. Although the deformation of the spherical stimuli differs greatly from (A) to (C), the resultant stress distributions and surface deflection at the finger pad are nearly identical.

Revisions in: Results: Experiment 1: computational modeling of the elasticity-curvature illusion

The old sentence: Specifically, in Fig 2A, stress distributions at the contact interface were nearly identical between the small-compliant (10 kPa-4 mm) and large-stiff (90 kPa-8 mm) spheres across all levels of load.

[p.7 line 142] The new sentence: Specifically, stress distributions at the epidermal-dermal interface were nearly identical between the small-compliant (10 kPa-4 mm) and large-stiff (90 kPa-8 mm) spheres across all levels of load (Fig 2A).

[p.7 line 147] The new Fig 2 caption: **Results of experiment 1: cues of cutaneous contact and proprioception.** (A) For the small-compliant (10 kPa-4 mm) and large-stiff (90 kPa-8 mm) spheres, stress distributions at the epidermal-dermal interface are nearly identical across all force loads. (B) Curves of stress distributions fairly well overlap for the 10 kPa-4 mm and 90 kPa-6 mm spheres. (C) Distinct stress distributions were obtained for spheres with the same elasticity but varied radii. (D) Proprioceptive cues of finger displacement are simulated for all force loads.

Old sentences: In addition to spatial distributions of stress, other deformation variables were also evaluated. The strain energy density (SED) and the deflection of the skin's surface were calculated and analyzed.

[p.8 line 153] New sentences: In addition to spatial distributions of stress, other response variables were also evaluated. The strain energy density (SED) at the epidermal-dermal interface and the deflection of the skin's surface were calculated and analyzed.

3. **Figures 1, 2, 3: It is not clear why a 90 kPA / 6mm stimulus is included in the four examples, rather than 90 kPA / 4 mm, which would mirror the other stimuli (all possible combinations of 10/90 kPa and 4/8mm). This should be fixed / explained.**

As shown in S1A Fig, either an increase of the elasticity (each row) or a decrease of the radius (each column) increased the stress/SED quantities. This resulted in two distinct sphere examples: the highest responses for the 90 kPa-4 mm sphere and the lowest for the 10 kPa-8 mm sphere. There is no essential difference between these two if taking either as a comparison to the other stimuli.

However, changes in the radii counteracted the changes in elasticity, resulting in nearly identical stress/SED distributions for the small-compliant (10 kPa-4 mm) and large-stiff (90 kPa-8 mm) stimuli. In addition, based on biomechanical measurements (Fig 5), the 90 kPa-6 mm stimulus can also generate similar contact areas with the aforementioned two stimuli. Besides, cutaneous cues of stress/SED distributions and surface deflection derived from the 90 kPa-6 mm were also overlapped with the two stimuli.

Therefore, we consider these three stimuli ([10,4], [90,6], and [90,8]) as the illusion case spheres that may generate indiscriminable cutaneous cues. Meanwhile, since there is no essential difference in terms of contrasting the illusion case spheres, we include only one of the two distinct stimuli, 10 kPa- 8 mm, as the comparison case. We changed a few sentences to better clarify this point.

Revisions in: Results: Experiment 1: computational modeling of the elasticity-curvature illusion

[p.7 line 129] The new sentence: Note that the 10 kPa-8 mm sphere was taken as the comparison case in the following analyses.

The old sentence: Similar to the results in Fig 2, SED distributions and skin surface deflection from the three spheres were nearly inseparable, …

[p.8 line 157] The new sentence: Similar to the results in Fig 2, SED distributions and skin surface deflection from the three spheres (10 kPa-4 mm, 90 kPa-6 mm, and 90 kPa-8 mm) were nearly inseparable, …

The old sentence: It indicates that in passive touch where only cutaneous cues are perceptible, one might be unable to differentiate the small-compliant and large stiff spheres.

[p.8 line 163] New sentences: It indicates that in passive touch where only cutaneous cues are perceptible, one might be unable to differentiate the aforementioned spheres. Therefore, these three stimuli (10 kPa-4 mm, 90 kPa-6 mm, and 90 kPa-8 mm) were denoted as the “illusion case spheres.”

4. **In the psychophysical results, performance is “significantly improved” in the passive condition with the inverse force rate. However, are any of the passive results actually different from baseline (50%)? If anything, it appears to me that the results in the passive inverse condition are closer to baseline than in the other passive condition. Similar to point 1) above, different force rates would also lead to different tactile feedback during the initial dynamic part of the contact.**

We changed the S3 Table (percentage of correctness), made the new S4 Table (sensitivity *d’*), and revised the text to better clarify the psychophysical results and tactile cues elicited during the dynamic contact, especially for the passive touch.

Compared with the “passive same” condition (46.1% ± 5.7, average *d’* = 0.42), performance was indeed improved in “passive inverse” condition (52.8% ± 6.7, average *d’* = 1.19). Specifically, correctness was improved by average of 13.33%, except for the last three pairs (S3 Table). Despite for the statistically significance, we agree that these two passive conditions yielded a chance performance (~50%). This aligns with prior work [15] indicating that in passive touch, discrimination correctness may be below 50% when force-rate cue is “inversely” applied to the stimuli: softer object was indented “inversely” at a higher force-rate and the harder object was indented at a lower force-rate.

For the “passive direct” condition, where force-rate cues were controlled to be directly proportional to the softness, discrimination correctness was significantly improved from the “passive same” ($U = 0.0$, $p < 0.0001$, $d = 4.33$) and “passive inverse” ($U = 0.5$, $p < 0.0001$, $d = 3.72$) condition. Indeed, this direct correlation between force-rate cue and softness has been found in active exploration [3,13]: we usually apply higher force-rate to explore the harder object. This indicates that, in agreement with the reviewer’s comment, the modulation of force-rate cue could evoke alternate perceptible inputs to discrimination. Specifically, we showed that the change rate of force can proportionally elicit the change of gross contact area (S5 Fig). While we cannot directly measure the rate of change of contact area in this work, based on this strong linear correlation between force and contact area, one could easily extrapolate this finding to the dynamic

contact phase by discretizing the terminal contact area/force into the instantaneous contact area/force throughout all contact stages (i.e., initial contact – ramp stage – plateau phase). In other studies using a 3D imaging approach [14,21], we indeed demonstrated that force-rate cue can proportionally elicit the change of contact area amidst dynamic contact. Furthermore, prior studies by Moscatelli, *et al.* explicitly demonstrated that the change of contact area of this kind can naturally induce a perceptible sensation of relative finger displacement in the stationary hand [16,19,22]. These findings render both theoretical and empirical evidences that can be applied herein: the improved discriminability in passive touch is likely augmented by the finger proprioception elicited by instantaneous changes of contact area, that is originally elicited by the direct force-rate cue. As a comparison, when force-rate cue is "inversely" applied to the stimuli, the elicited cutaneous cue would be evitability contraindicated to the perceived softness, eventually confound participants in discrimination.

Revisions in: Experiment 3: psychophysical evaluation of the elasticity-curvature illusion

[p.13 line 281] New sentences: <u>However, this discrimination correctness (with all participants aggregated) was significantly higher compared with the "passive same force-rate" condition (U = 24.0, $p < 0.05$, d = 1.03). Average d' value of 1.19 across stimulus pairs also indicated an improved, but still poor discrimination sensitivity under this condition (detailed in S4 Table). This aligns with prior work demonstrating that participants exhibit a chance performance (~50%) when force-rate cue is "inversely" applied in passive touch [26].</u>

[p.13 line 293] New sentences: <u>These results empirically validate that, when force-rate cues are "directly" applied during the contact, cues besides those cutaneous become available in discriminating the illusion case spheres. It further indicates that the controlled force-rate cues may elicit alternate perceptible inputs and are likely perceived akin to proprioception, a point which will be detailed in the Discussion.</u>

Revisions in: Discussion: Change of cutaneous contact as a cue to proprioception

[p.16 line 354] The new sentence: <u>We hypothesize that the modulation of force under "passive direct/inverse force-rate" condition – where the softer stimulus was indented "directly/inversely" at a lower/higher force-rate than the harder stimulus – provides an alternate perceptible input during the dynamic contact phase, also tied to finger proprioception.</u>

[p.16 line 358] New sentences: <u>While we cannot directly measure the rate of change of contact area, due to the limitations of the ink-based method only being able to measure terminal contact area, one could easily extrapolate this correlation to the dynamic contact phase by discretizing the terminal contact area/force into the instantaneous contact area/force. Using the 3D imaging technique, we indeed demonstrated that force-rate cue can proportionally elicit the instantaneous change of contact area [44]. Such cues might therefore induce the illusion of fingertip displacement amidst dynamic contact [37,45].</u>

[p.16 line 369] The new sentence: Therefore, when passively exploring the illusion case spheres under the modulation of force-rate, the improved discriminability is likely derived from the proprioceptive sensation elicited by the change of contact area, which is originally induced by the force-rate cue.

References

3. Xu C, He H, Hauser SC, Gerling GJ. Tactile exploration strategies with natural compliant objects elicit virtual stiffness cues. IEEE Trans Haptics. 2020;13: 4–10. doi:10.1109/TOH.2019.2959767
13. Xu C, Hauser SC, Wang Y, Gerling GJ. Roles of force cues and proprioceptive joint angles in active exploration of compliant objects. 2019 IEEE World Haptics Conference (WHC). IEEE; 2019. pp. 353–358. doi:10.1109/WHC.2019.8816159
14. Li B, Hauser S, Gerling GJ. Identifying 3-D spatiotemporal skin deformation cues evoked in interacting with compliant elastic surfaces. IEEE Haptics Symposium, HAPTICS. IEEE Computer Society; 2020. pp. 35–40. doi:10.1109/HAPTICS45997.2020.ras.HAP20.22.5a9b38d8
15. Hauser SC, Gerling GJ. Force-rate cues reduce object deformation necessary to discriminate compliances harder than the skin. IEEE Trans Haptics. 2018;11: 232–240. doi:10.1109/TOH.2017.2715845
16. Moscatelli A, Bianchi M, Serio A, Terekhov A, Hayward V, Ernst MO, et al. The change in fingertip contact area as a novel proprioceptive cue. Curr Biol. 2016;26: 1159–1163. doi:10.1016/j.cub.2016.02.052
19. Moscatelli A, Bianchi M, Serio A, Al Atassi O, Fani S, Terekhov A, et al. A change in the fingertip contact area induces an illusory displacement of the finger. Lecture Notes in Computer Science (including subseries Lecture Notes in Artificial Intelligence and Lecture Notes in Bioinformatics). Springer Verlag; 2014. pp. 72–79. doi:10.1007/978-3-662-44196-1_10
21. Hauser SC, Gerling GJ. Imaging the 3-D deformation of the finger pad when interacting with compliant materials. IEEE Haptics Symposium, HAPTICS. IEEE; 2018. pp. 7–13. doi:10.1109/HAPTICS.2018.8357145
22. Moscatelli A, Bianchi M, Ciotti S, Bettelani GC, Parise C V., Lacquaniti F, et al. Touch as an auxiliary proprioceptive cue for movement control. Sci Adv. 2019;5: eaaw3121.
