# Supplementary material for: An elasticity-curvature illusion decouples cutaneous and proprioceptive cues in active exploration of soft objects
Source: PLoS Comput Biol. 2021 Mar 22;17(3):e1008848. doi: 10.1371/journal.pcbi.1008848 (PMC8016306; doi:10.1371/journal.pcbi.1008848)
Supplement: Attachment — Submitted filename: ResponseToReviewers_PCOMPBIOL-D-20-01703R2-Responses.pdf [file pcbi.1008848.s015.pdf]

Reviewer #1 Comments

We thank the reviewer for the detailed and valuable suggestions. We changed the text accordingly to better articulate our experimental results and general findings.

1. **Line 19: constrained stationery – constrained to be stationery**

   [p.2 line 19] The new sentence: …when the finger is constrained to be stationary...

2. **Line 20: are made perceptible – are perceptible**

   [p.2 line 20] The new sentence: …from mechanosensitive afferents are perceptible.

3. **Line 25: cutaneous contact – cutaneous contact cues**

   [p.2 line 25] The new sentence: … inputs amidst indiscriminable cutaneous contact cues.

4. **Line 46: comparing to prior experiences – comparing what?**

   We learned from Neisser [1] and prior studies [2,3] that perception is a repetitive cycle combining prior expectations, environmental explorations and resulting sensory inputs, and the updating of internal representation. Therefore, within this perceptual cycle, we compare current percepts of the object with our prior expectations or experiences of this particular object (or similar ones), and thus, update our final perception.

1. Neisser U. Cognition and reality: Principles and implications of cognitive psychology. W H Freeman/Times Books/ Henry Holt & Co; 1976.
2. Xu C, He H, Hauser SC, Gerling GJ. Tactile exploration strategies with natural compliant objects elicit virtual stiffness cues. IEEE Trans Haptics. 2020;13: 4–10. doi:10.1109/TOH.2019.2959767
3. Xu C, Gerling GJ. Time-dependent Cues Encode the Minimum Exploration Time in Discriminating Naturalistic Compliances. IEEE Haptics Symposium, HAPTICS. 2020. pp. 22–27. doi:10.1109/HAPTICS45997.2020.ras.HAP20.7.ec43f6a7

   [p.3 line 46] The new sentence: We seamlessly do so by recruiting sensorimotor inputs, fine-tuning motor control strategies, comparing current percepts to our prior expectations, and updating internal representations [8].

5. **Lines 57-58: could be perceived as being convex or concave curved – can be perceived as being convex or concave (remove "curved")**

   [p.3 line 58] The new sentence: … perceived as being convex or concave [9,14].

6. **Lines 61-63: Explain the task in more detail this is very cryptic**

   [p.4 line 61] New sentences: …misperception in speed by touch [15]. In particular, observers are asked to estimate the speed of a moving belt stimulus. Compared with tracking the stimulus with a guided arm movement, where the finger is moving along with the belt's motion (i.e., proprioception is available), observers can overestimate the stimulus speed by touching the stimulus with a stationary hand (i.e., tactile cues only).

7. **Lines 67-68: This should be referred to as the pseudo-haptic effect**

We provided a better example to illustrate this point.

[p.4 line 69] The new sentence: …e.g., the “size-weight” illusion could be exploited to create particular stimuli in virtual reality whose physical properties may be perceived as changing during interactions.

8. **Line 85: What are non-distinct cutaneous cues?**

The non-distinct cutaneous cues are mostly the interior stress distributed at the epidermal-dermal interface and gross contact areas upon finger pad-to-stimulus contact.

In passive touch, our finite element model predicts that small-compliant (10 kPa-4 mm) and large-stiff (90 kPa-8 mm) spheres will generate similar cutaneous contact cues of interior stress (Fig 2A), strain energy density (Fig 3A), and surface deflection (Fig 3C). Furthermore, our biomechanical measurements show that these spheres can also generate non-distinct gross contact areas.

[p.5 line 87] The new sentence: …when the finger is stationary and <u>only non-distinct cutaneous cues of interior stress and gross contact areas</u> are available for perception.

9. **Lines 162-163: therefore naturally dissociate from proprioceptive movements – is the point that the cutaneous cues are similar and therefore non-informative whereas proprioceptive cues may yield useful information about contact?**

Yes, indeed. We changed the text to better clarify this point.

[p.8 line 163] The new sentence: These results demonstrate that small-compliant and large-stiff stimuli can generate nearly identical cutaneous contact cues, <u>therefore, non-informative for discriminating compliances whereas proprioceptive cues may be useful.</u>

10. **Line 196: profile – profiles**

[p.9 line 199] The new sentence: …generate similar <u>cutaneous contact profiles.</u>

11. **Line 234: no significantly difference – no significant difference (also on lines 249, 311)**

We modified associated language throughout the manuscript.

[p.11 line 237] The new sentence: … overlapped (<u>no significant difference detected</u>) …

[p.11 line 252] The new sentence: … overlapped <u>(no significant difference detected),</u>

[p.14 line 313] The new sentence: …cues <u>(no significant difference detected)</u> …

[p.15 line 336] The new sentence: … evoke <u>significant differences</u> in fingertip…

12. **Line 269: Meanwhile… - this implies that while the first task was occurring something else was being implemented which was not the case – remove meanwhile (also line 391)**

[p.12 line 271] The new sentence: To further investigate the utility of temporal cues …

[p.17 line 395] The new sentence: As it is difficult to measure the material properties …

13. **Line 274: yielded a chance performance – the d' value indicates that performance is at chance is does not yield it**

[p.12 line 276] The new sentence: The mean *d'* of 0.42 indicated a chance…

14. **Line 282: discrimination correctness – "percentage of correct responses" seems a better term than discrimination correctness, average percentage of correctness and result of correctness (all in several places, lines 350-352).**

We agree and changed the text throughout the manuscript.

[p.12 line 274] The new sentence: … stimuli (percentage of correct responses: ...

[p.13 line 283] The new sentence: … with a percentage of correct responses of 52.8% ± 6.7. However, this result (with all participants aggregated) was significantly higher…

[p.13 line 291] The new sentence: … spheres near a 75% threshold (76.7% ± 5.4). This percentage of correct responses (with all participants aggregated) …

[p.14 line 304] The new sentence: … with a percentage of correct responses …

[p.14 line 306] The new sentence: … significantly better discrimination performance …

[p.16 line 353] The new sentence: … with a percentage of correct responses of …

15. **Line 283 and elsewhere in the Results section: I assume the "averages" reported are means? If so, they should be referred to as means (or modes or medians).**

Yes, we reported means in the Results. We changed the text throughout the manuscript.

[p.8 line 162] The new sentence: … mean values of cutaneous responses over…

[p.11 line 234] The new sentence: … (mean contact area: 0.90 ± 0.12 cm^2, mean ± SD), while the others were significantly distinct from them (mean contact area: ...

[p.11 line 250] The new sentence: … the mean contact area for the three illusion cases is 0.87 ± 0.10 cm^2 while the other distinct stimulus derived a mean contact area of...

[p.12 line 276] The new sentence: The mean *d'* of 0.42 indicated a chance…

[p.13 line 285] The new sentence: The mean *d'* value of 1.19 across …

[p.14 line 305] The new sentence: …and a mean sensitivity of 3.53 ...

[p.25 line 564] The new sentence: ...set as the mean value of the data normalized, …

16. **Line 319: specifically where – specifically the situation in which**

[p.14 line 321] The new sentence: …specifically the situation in which small-compliant…

17. Line 325: cues akin to proprioception – why are the cues "akin"?

[p.15 line 327] The new sentence: … when proprioceptive cues augment…

18. Line 332: "of touch force was volitionally controlled to be non-distinct among the illusion case": using the word volitionally in this context does not seem appropriate – the touch force was controlled but it is not clear that participants were consciously aware that they were deliberately controlling this force which is what the concept of volition entails.

Agreed. We changed the text accordingly. In Experiment 3, participants were instructed to explore the stimuli under fully active, behavioral control of their touch force. They were explicitly instructed to press into the stimulus with a force load of 2 N. A sound alarm served as real-time feedback when the touch force reached 2 N.

[p.12 line 266] The new sentence: …force-rate cues are behaviorally applied.

[p.13 line 301] The new sentence: …under fully active, behavioral sensorimotor control.

[p.15 line 332] The new sentence: …participants behaviorally control the exploratory...

[p.15 line 334] The new sentence: …of touch force was behaviorally controlled...

[p.15 line 344] The new sentence: …echoes theories of active, behavioral control...

[p.17 line 398] The new sentence: …exploratory strategy of behaviorally controlling...

[p.24 line 552] The new sentence: …with a terminal touch force of 2 N. When their...

19. Line 345: improve – improves (the availability….improves)

[p.15 line 347] The new sentence: … the availability of force-related cues improves…

20. Line 377: This comes in general agreement – what comes?

[p.17 line 380] The new sentence: These findings come in general agreement…

21. Line 446: in attempt -in an attempt

[p.20 line 450] The new sentence: …were simulated in an attempt to approximate …

22. Line 471: Velcro straps were further used to constrain if any slipperiness was detected. – constrain what? The forearm?

We change the text to better clarify this point.

[p.21 line 475] The new sentence: … were further used to constrain the forearm …

Reviewer #2 Comments

General Comments:

I carefully checked through the authors changes. They really did a good job in responding to my concerns. From my side, there is nothing to quibble about. Hence, I recommend acceptance. Only a minor point: Maybe the author might want to recheck their Mann-Whitney U-statistics, because it quite often was 0.

We thank the reviewer for the valuable comments. Those revisions suggested by the reviewer indeed help us to better clarify our experimental procedures and results.

Proactively, we revisited all the Mann-Whitney U tests. In general, we had applied this test to two groups of data to evaluate the difference between the two groups. The *U* statistic of 0 indicates that all of the values from one group are strictly greater than all the values of another group. This indicates an extreme situation where we can still reject the null hypothesis that the values have been drawn from the same distribution. The effect size value also supports this conclusion. In particular, we obtained the *U* statistic of 0 in four places:

[p.11 line 233; p. 11 line249; p.12 line 255] As shown in Fig 5A, 5B, and 5D, all contact areas of the distinct sphere (10 kPa-8 mm) were larger than the illusion case spheres.

[p.13 line 293] As shown in Fig 6A, all discrimination results of the “passive direct force-rate” task were larger than the ”passive same force-rate” task.

Besides, we corrected one of the reported effect size values as follow. The updated effect size still indicates that this significant result has a large effect.

[p.12 line 255] The new sentence: Specifically, there was a significant difference between the contact areas of the illusion case and distinct spheres across all force levels (U = 0.0, p < 0.0001, $\underline{d = 4.94}$).

Reviewer #3 Comments

General Comments:

All my concerns have been addressed. I would like to thank the authors for providing manuscript changes within the rebuttal, making it very easy to evaluate changes. I look forward to seeing the paper in print!

We thank the reviewer for the constructive and valuable suggestions. The further analysis on computational modeling suggested by the reviewer indeed helps us to better elaborate the results and articulate the overall findings.
